# E2F signature is predictive for the pancreatic adenocarcinoma clinical outcome and sensitivity to E2F inhibitors, but not for the response to cytotoxic-based treatments

**DOI:** 10.1038/s41598-018-26613-z

**Published:** 2018-05-29

**Authors:** Wenjun Lan, Benjamin Bian, Yi Xia, Samir Dou, Odile Gayet, Martin Bigonnet, Patricia Santofimia-Castaño, Mei Cong, Ling Peng, Nelson Dusetti, Juan Iovanna

**Affiliations:** 1 Centre de Recherche en Cancérologie de Marseille (CRCM), INSERM U1068, CNRS UMR 7258, Aix-Marseille Université and Institut Paoli-Calmettes, Parc Scientifique et Technologique de Luminy, Marseille, France; 2 Aix-Marseille Université, CNRS, Centre Interdisciplinaire de Nanoscience de Marseille, UMR 7325, «Equipe Labellisée Ligue Contre le Cancer», Marseille, France; 3 Chongqing Key Laboratory of Natural Product Synthesis and Drug Research, School of Pharmaceutical Sciences, Chongqing University, Chongqing, China

## Abstract

The main goal of this study was to find out strategies of clinical relevance to classify patients with a pancreatic ductal adenocarcinoma (PDAC) for individualized treatments. In the present study a set of 55 patient-derived xenografts (PDX) were obtained and their transcriptome were analyzed by using an Affymetrix approach. A supervised bioinformatics-based analysis let us to classify these PDX in two main groups named E2F-highly dependent and E2F-lowly dependent. Afterwards their characterization by using a Kaplan-Meier analysis demonstrated that E2F high patients survived significantly less than E2F low patients (9.5 months vs. 16.8 months; p = 0.0066). Then we tried to establish if E2F transcriptional target levels were associated to the response to cytotoxic treatments by comparing the IC50 values of E2F high and E2F low cells after gemcitabine, 5-fluorouracil, oxaliplatin, docetaxel or irinotecan treatment, and no association was found. Then we identified an E2F inhibitor compound, named ly101-4B, and we observed that E2F-higly dependent cells were more sensitive to its treatment (IC50 of 19.4 ± 1.8 µM vs. 44.1 ± 4.4 µM; p = 0.0061). In conclusion, in this work we describe an E2F target expression-based classification that could be predictive for patient outcome, but more important, for the sensitivity of tumors to the E2F inhibitors as a treatment. Finally, we can assume that phenotypic characterization, essentially by an RNA expression analysis of the PDAC, can help to predict their clinical outcome and their response to some treatments when are rationally selected.

## Introduction

Pancreatic ductal adenocarcinoma (PDAC) is a mortal disease characterized by an expected survival ranging from few as 3 months to, although infrequently, more than 5 year after its diagnosis^1^. The causes inducing this variability remain unfortunately poor known and virtually unstudied. Moreover, response to the standard treatments is also variable with a global objective response to gemcitabine and Folfirinox, the two standard protocols used for treating patients with a PDAC, of only 10^2^ and 31%^3^ respectively. The variability in this response seems to be due, on one hand, to the difficult for drugs to reach the transformed cells because the compact stroma, characteristic of the PDAC, results in a few vessels formation and, on the other hand and most importantly, to the strong differences in cellular susceptibility to drugs into tumors.

A model of PDAC development proposes a genetic-based progressive disease that was inspired on the model postulated by Fearon and Vogelstein several years ago for colon cancer^4^. This model includes the early low-grade pancreatic lesions PanIN1A, PanIN1B, PanIN2 and the high-grade PanIN3, PDAC and lastly its metastasis in a progressive and continuous manner^5,6^. This model is almost exclusively genetic and it is based on the fact that activating mutations in the *KRAS* oncogene is nearly universal in human PDAC, and targeting of mutated *KRAS* to mouse pancreatic progenitors recapitulates the human PanIN-to-PDAC progression sequence^7^. The hypothesis is that high-grade lesions develop upon accumulation of further mutational events, mainly involving inactivation of other tumor suppressors such as *INK4A*, *TP53*, *SMAD4*, *ARID1A* or *BRCA2*^5^. Several hundred of additional somatic mutations were also found in advanced PDAC with a variable incidence^8,9^. Even if this model could partially explain the PDAC as a progressive disease, it cannot elucidate their variable phenotype, their very different clinical outcome as well as their inconstant response to treatments. On the contrary, classification of PDAC based on their transcriptome seems to be distinctive and permits to distinguish among patients with bad or better prognosis^8,10,11^ or between patients that are responders or not to some drugs such as BETi for MYC high patients as recently reported by us^12^. Because the transcriptomic analysis of the PDAC let to make a clinically relevant classification whereas the genetic-based criteria did not, we assume that phenotypic differences in the PDAC, with consequences on their clinical outcome and responses to treatments, appear to be regulated at the post-genetic level. Said in other words, it is probably that mutations on master genes induce the pancreatic transformation, while the behavior of the PDAC is largely modulated at post-genetic levels such as epigenetic of transcriptional levels. Therefore it sounds reasonable to study the PDAC at transcriptomic instead genetic levels when we were interested in detecting therapeutically targetable intracellular pathways.

In the present study we obtained a set of 55 patient-derived xenografts (PDX) and analyzed their transcriptome by using an Affymetrix approach. A supervised bioinformatics-based analysis of the transcriptional E2F targets let us to classify these PDX in two main groups named E2F-highly dependent and E2F-lowly dependent. The clinical relevance revealed that E2F signature is able to predict the clinical outcome but not its sensitivity to cytotoxic anticancer drugs. In addition, we identified an E2F inhibitor compound and demonstrated, as expected, that E2F-higly dependent cells are more sensitive to this drug candidate.

*E2F* is a group of genes that codifies a family of transcription factors in higher eukaryotes. The E2F family of transcription factors bind to the typical E2F motif (TTTCGCGC or slight variations of this sequence)^13^ that exists in many genes involved in DNA synthesis, cell cycle progression and mitosis^14^. Indeed *in vivo* studies indicate that the roles and regulation of these factors are complex; E2F1-3 are most commonly associated with transcriptional activation of genes involved in normal cell cycle transitions, where their activities are restrained by their association with RB family members in a manner that is relieved by CDK-mediated hyperphosphorylation of RB^15^. E2F4 and E2F5 are most strongly linked to transcriptional repression during quiescence^16^, whereas E2F6 has been linked to polycomb-mediated gene regulation^17^. E2F7/8 are transcriptional repressors with an atypical structure, having two DNA-binding domains and lacking a dimerization domain, which is required for association with dimerization partner proteins that appear to be important for the sequence-specific binding capacity of other E2Fs^18,19^.

## Results

### Patients derived xenografts as a model for identifying functionally related PDAC

We developed a strategy by which virtually all PDAC can be studied from samples obtained from surgery and from EUS-FNA growth as PDX, avoiding a selection bias when including exclusively surgical samples. In fact, using our strategy we obtained 100% of the surgery-derived PDX and around 80% when derived from EUS-FNA, showing that virtually all tumors are studied^20^. PDX, as developed by us, is a whished model of study PDAC by at least two main reasons. The first one is the fact that human PDAC contains a variable amount (from 15 to 85%) of stroma that is significantly reduced when it is growing on mice, and the second is that only around of 15% of patients are operated but essentially all the patients were biopsied. In other words, using our approach we may study predominantly the transformed cells from virtually all patients. In this study we analyzed 55 consecutives PDX obtained as described above. Other study, use PDX as PDAC tumor avatars as well as cell lines derived from PDX. Authors clearly shows that patients derived models are representing the heterogeneity of PDAC and exhibits the same mutational landscape and transcriptomic profile than the original tumor. Low passages PDX models are clustered together with the primary tumor in unsupervised analysis showing a good picture of the primary tumor phenotype^21^.

### Unsupervised transcriptome analysis reveals two clusters of PDAC

**Figure 1:**
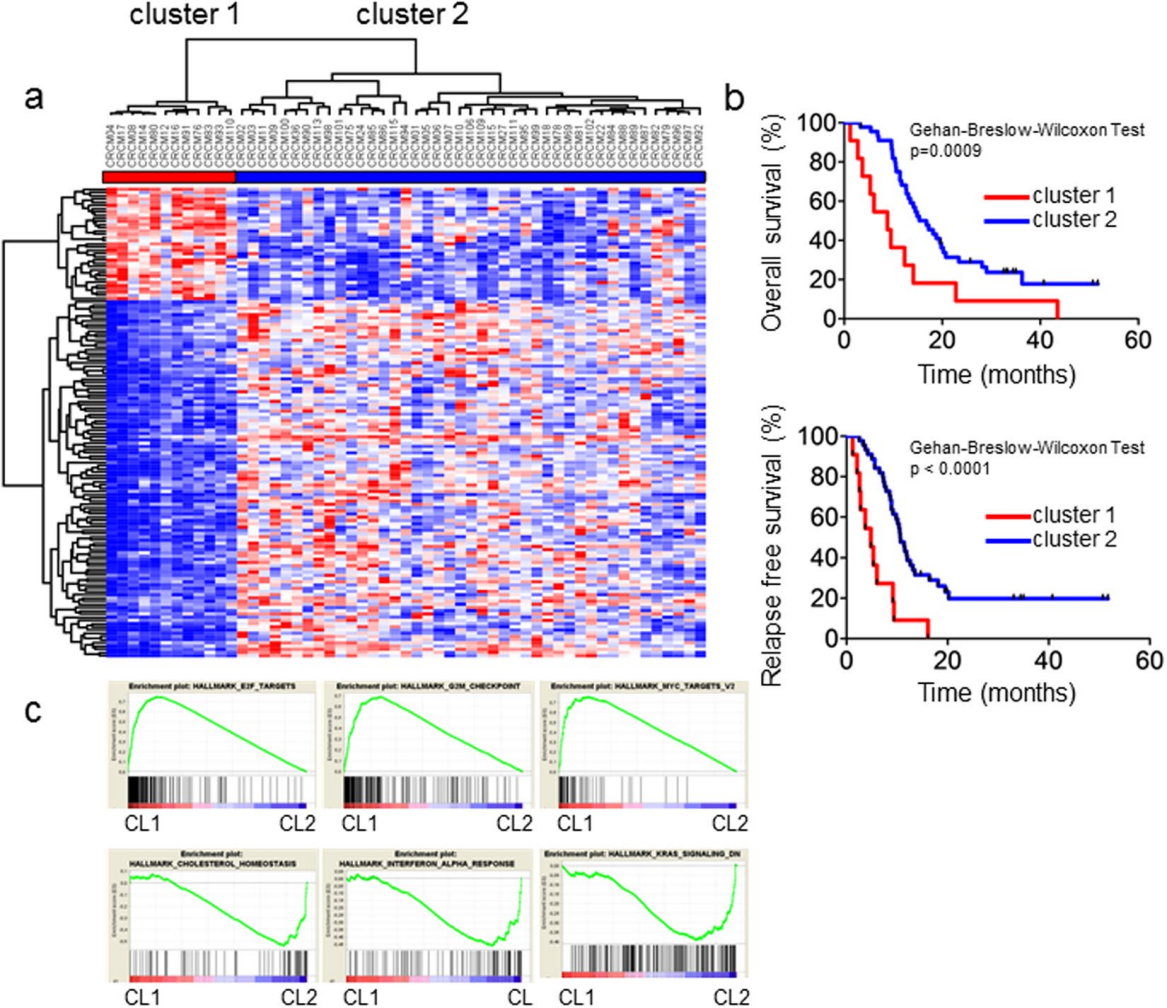
Identification of a PDAC patient’s subgroup associated with short relapse and overall survival by Affymetrix approach. (**a**) Hierarchical clustering and expression heatmap analyzed by a non‐supervised method. Two majors clusters were defined by differential expression patterns (cluster 1 n = 12 versus cluster 2 n = 43). RMA normalized gene expression is represented in color to indicate relative gene expression (high in red, low in blue). (**b**) Kaplan–Meier curves showing the overall (upper graph) and relapse‐free survival (lower graph) for cluster 1 and cluster 2 subgroups. The *p‐*values were calculated using Gehan-Breslow-Wilcoxon Test with GraphPad v5.0 software. (**c**) GSEA analysis showing the top six biological process that differ the most between the two subgroups. Fifty gene set relative to the Hallmarks repertory from Molecular Signature DataBase (MsigDB) were analyzed.

RNA from 55 PDX was hybridized on Affymetrix arrays in duplicate. An unsupervised hierarchical clustering of the data obtained by an Euclidian distance, after the replicative averaging, of the 110 transcriptome (55 patients in duplicate, the correlation matrix of samples is provided in Supplementary Table 1) reveals 2 main groups of PDAC, one containing 12 patients (cluster 1) and the other one contains the 43 remaining (cluster 2) (Fig. 1a). A total of 146 transcripts were identified as differentiating significantly both groups using four statistical parameters (raw p-value < 0.002, FDR adjust p-value < 0.05, t-test score >6 for overexpressed genes and ≤−6 for downregulated genes and fold change >1.2 and ≤−1.2 (see Supplementary Table 2). Thirty-five transcripts were overexpressed in PDX from cluster 1 whereas 111 are overexpressed in PDX from cluster 2. Interestingly, as demonstrated in Fig. 1b, Kaplan-Meier analysis over the 55 patients grouped by these clustering reveals that patients from cluster 1 survived significantly less than patients from group 2 (8.8 months vs. 16.8 months; p-value of 0.0009). Moreover, we observed a significant difference on the relapse-free survival (RFS) between patients corresponding to these clusters (4.8 months vs. 10.7 months; p-value < 0.0001). Finally, we studied the enrichment of intracellular pathways within each PDAC cluster using GSEA. To do this, we proceeded to GSEA analysis to the entire transcriptome data of the 55 PDX. We found that whereas the pathways corresponding to hallmark_E2F_targets (FDR =<10^−3^), hallmark_MYC_targets_V1 (FDR =<10^−3^); hallmark_G2M_checkpoint (FDR =<10^−3^) were enriched in cluster 1, the pathways corresponding to the hallmark_CHOLESTEROL_homeostasis (FDR =<10^−3^), hallmark_INTERFERON_ALPHA_response (FDR =<10^−3^), hallmark_KRAS_signaling_DN (FDR =<10^−3^) were enriched in cluster 2 as showed in Fig. 1c. To further investigate the possible implication of E2F factors in the cluster 1 we analyzed the transcription factor (TF) motifs present on promoters regions within differentially expressed genes. We observed that most of them share a common E2F family binding motif (Supplementary Table 3). Moreover, using Ingenuity pathway analysis, we observed that E2F3 is part of the top upstream activated transcriptional regulator and may be responsible for the expression change between the two subgroups (Supplementary Figure 1). Altogether, these data indicate significantly functional differences between these groups and supporting that transcriptomic analysis is useful for relevant classification. Clinically, this observation is opening on the selection of patients to use specific treatments depending of the biological characteristics of their tumor.

### Supervised transcriptome analysis reveals E2F-highly dependent and E2F-lowly dependent clusters of PDAC

**Figure 2:**
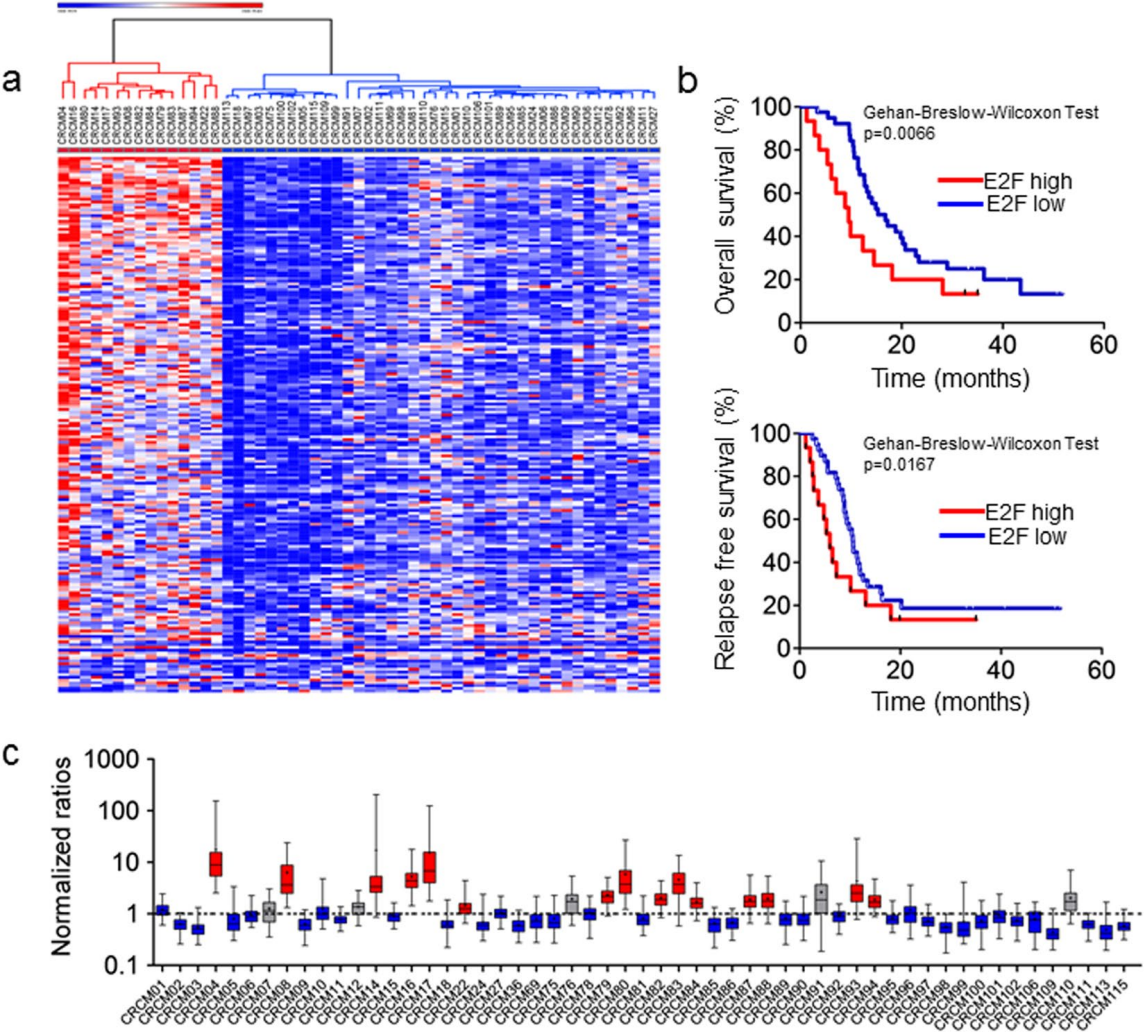
Development of an E2F-dependant transcriptional signature for classifying PDAC patients. (**a**) Heatmap representing the expression patterns of the 196 selected E2F transcriptional targets from MsigDB between the two clusters of PDAC patients. The lines corresponding to the genes were rank-ordered from the most differentially expressed transcripts to the less differentially expressed transcripts. The color coded expression values were presented as in Fig. 1a. (**b**) Kaplan–Meier curves showing the overall (upper graph) and relapse‐free survival (lower graph) for E2F high and E2F low subgroups. The *p‐*values were calculated as in Fig. 1b. (**c**) Box plots representing the normalized expression ratios for the twenty-four selected transcripts in the E2F‐dependant transcriptional signature. The black dotted line shows ratios equal to 1. Ratios >1 indicate an E2F‐high profile, in red, and ratios <1 correspond to E2F‐low profile, in blue. The greys colored box plots indicate the five false positives detected with the signature (duplicates [2 chips/PDX]).

Then, because the E2F activated pathway appears as a classifier of PDAC, we tried to select all the patients with a significant high E2F target expression level. Therefore, we selected a set of 196 transcripts (see Supplementary Table 4) which expression is dependent of E2F transcriptional activity from the Molecular signatures database (MSigDB) 3.0.^22^ and performed a supervised clustering on the same set of PDX. Using this clustering criteria we observed that 4 PDX (CRCM12, CRCM91, CRCM76 and CRCM110) switched from cluster 1 to E2F low and 7 PDX switched from cluster 2 to E2F high (CRCM22, CRCM79, CRCM82, CRCM84, CRCM87, CRCM88 and CRCM94) indicating a more precise E2F-dependency classification as showed in Fig. 2a.

Then, we studied the overall survival and RFS on the 55 patients by using a Kaplan-Meier analysis over these patients clustered as E2F high and E2F low respectively (Fig. 2b). We observed that E2F high patients survived significantly less than E2F low patients (9.5 months vs. 16.8 months; p-value of 0.0066). Moreover, we observed a significant difference on the RFS between patients corresponding to these clusters (6.0 months vs. 10.6 months; p-value of 0.0167). Altogether, these results suggest that classifying PDAC patients based on their E2F transcriptional cell cycle related target levels would have a clinical interest. Moreover, we screened the expression of all E2F’s isoforms in both subgroups. Interestingly, E2F’s isoforms are significantly upregulated in the E2F-high subgroup (Supplementary Figure 2).

### A 24-genes signature can discriminate between E2F-highly dependent and E2F-lowly dependent groups of PDAC

As a proof of concept to select patients with E2F high and E2F low activity we tried to select patients by using a small RNA signature (Table 1). Eleven transcripts (BIRC5, LMNB1, POLA2, DEPDC1, MCM2, CDK1, PTTG1, CDC20, PLK1, KPNA2 and AURKA) that are overexpressed in E2F high PDX and 13 mRNA (RHBDL2, DLEU7-AS1, TMEM63A, IGSF9, NEIL1, BDKRB2, PDZK1IP1, ERN2, CTSE, VSIG2, BCL2L15, LOC100505633 and TXNIP) that were, on the contrary, down-regulated in the same PDX were selected (for each transcript the raw p-values were < 0.002, FDR adjusted p-values < 0.05, t-test score > 6 and ≤−6 and the fold change were >1.2 and ≤−1.2)(see Supplementary Figure 4). The concept was that when a transcript was activated as it is expected in E2F high PDX, the ratio of up/down must be >1. Conversely, in PDX from patients with an E2F low activity this ratio must correspond to <1. To do this we proceeded to their normalization as follow: first, the sum of the expression values of all the patients of each up-regulated gen (e.g. for gen a: P1a + P2a + P3a + … P55a) correspond to arbitrarily 100. In the same way, we normalized expression values of the down-regulated genes (e.g. for gen A: P1A + P2A + P3A + … P55A) corresponding to arbitrarily 100. After that normalization, in each patient, we calculated the ratio between each up-regulated gene and each of the down-regulated transcript as follow (a/A, a/B, a/C, a/N), (b/A, b/B, b/C, b/N), etc. We considered that PDAC correspond to E2F high when the median of these ratios were over 1, but whereas when the median of these ratios were less than 1, we considered that the sample corresponded to E2F low. In Fig. 2c are represented all the 55 patients and the strategy seems to be efficient since all the E2F high patients (in red) are well differentiated from the E2F low (in blue). Only 5 patients resulted in false positive (in gray). Finally, we validated the 24-genes E2F signature in external cohort of PDAC from the TCGA consortium. From the original signature composed by 24 markers, data weren’t available for three markers (LOC100505633, DLEU7-AS1, and RHBDL2). The 24-genes signature is able to discriminate two clusters of patients named respectively TCGA E2F high and TCGA E2F low. Surprisingly, the DFS and OS of the 17 TCGA E2F high patients are reduced compared to the TCGA E2F low subgroup (Supplementary Figure 3).

**Table 1:** List of biomarkers used in the transcriptomic signature.

Genes overexpressed in cluster 1
BIRC5
LMNB1
POLA2
DEPDC1
MCM2
CDK1
PTTG1
CDC20
PLK1
KPNA2
**Genes downregulated in cluster 1**
AURKA
RHBDL2
DLEU7-AS1
TMEM63A
IGSF9
NEIL1
BDKRB2
PDZK1IP1
ERN2
CTSE
VSIG2
BCL2L15
LOC100505633
TXNIP

### E2F genes target level does not predicts sensitivity to cytotoxic drugs *in vitro*

**Figure 3:**
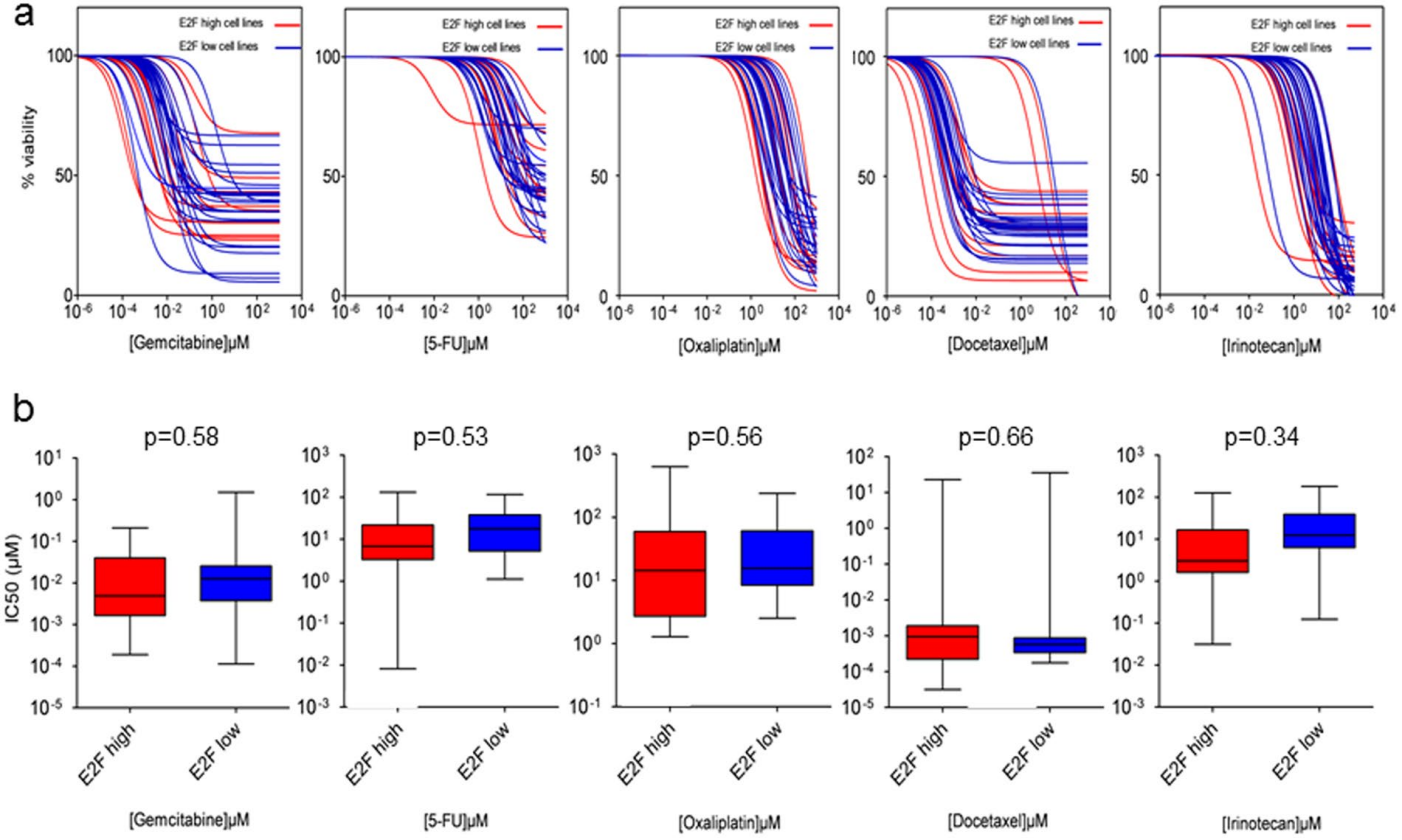
The E2F transcriptional signature doesn’t predict the PDAC sensitivity to cytotoxic drugs *in vitro*. (**a**) Chemograms assays showing the sensitivity of the E2F high cell lines (in red) and E2F low cell lines (in blue) to five chemotherapeutics drugs commonly using in clinic (gemcitabine, 5-FU, oxaliplatin, docetaxel and irinotecan). The concentrations of each drug vary from 1 nM to 1 M (**b**) Box plots representing the IC50 values were calculated using nonlinear regression of the log([drug]) versus normalized curve with robust fit shown in a.

Then, we tried to establish whether or not the E2F targets level was associated to the response to the chemotherapeutic treatments. A few studies have shown the potential implication of E2F targets or E2F isoforms up-regulation in drug resistance acquisition particularly to gemcitabine^23^ and 5-FU^24^ and oxaliplatin^25^. Our first hypothesis was that E2F highly dependent patients are more resistant to cytotoxic drugs. To test this question, we prepared 36 cell lines derived from the PDX as previously described^26^ and treated these cells with increasing concentrations of cytotoxic drugs such as gemcitabine, 5-fluorouracil, oxaliplatin, docetaxel and irinotecan (from 0.001 to 1000 mmol/L) and their sensitivity measured to obtain their IC50 characterizing each patient. Using this approach we were able to measure their relative chemosensitivity in both E2F high (red) and E2F low (blue) cells as showed in Fig. 3a. Then, we compared the IC50 of the E2F high and E2F low cells and found no correlation between both E2F targets level and sensibility to the cytotoxicity as showed in Fig. 3b.

### Identification of ly101-4B compound as an inhibitor of the E2F activity

**Figure 4:**
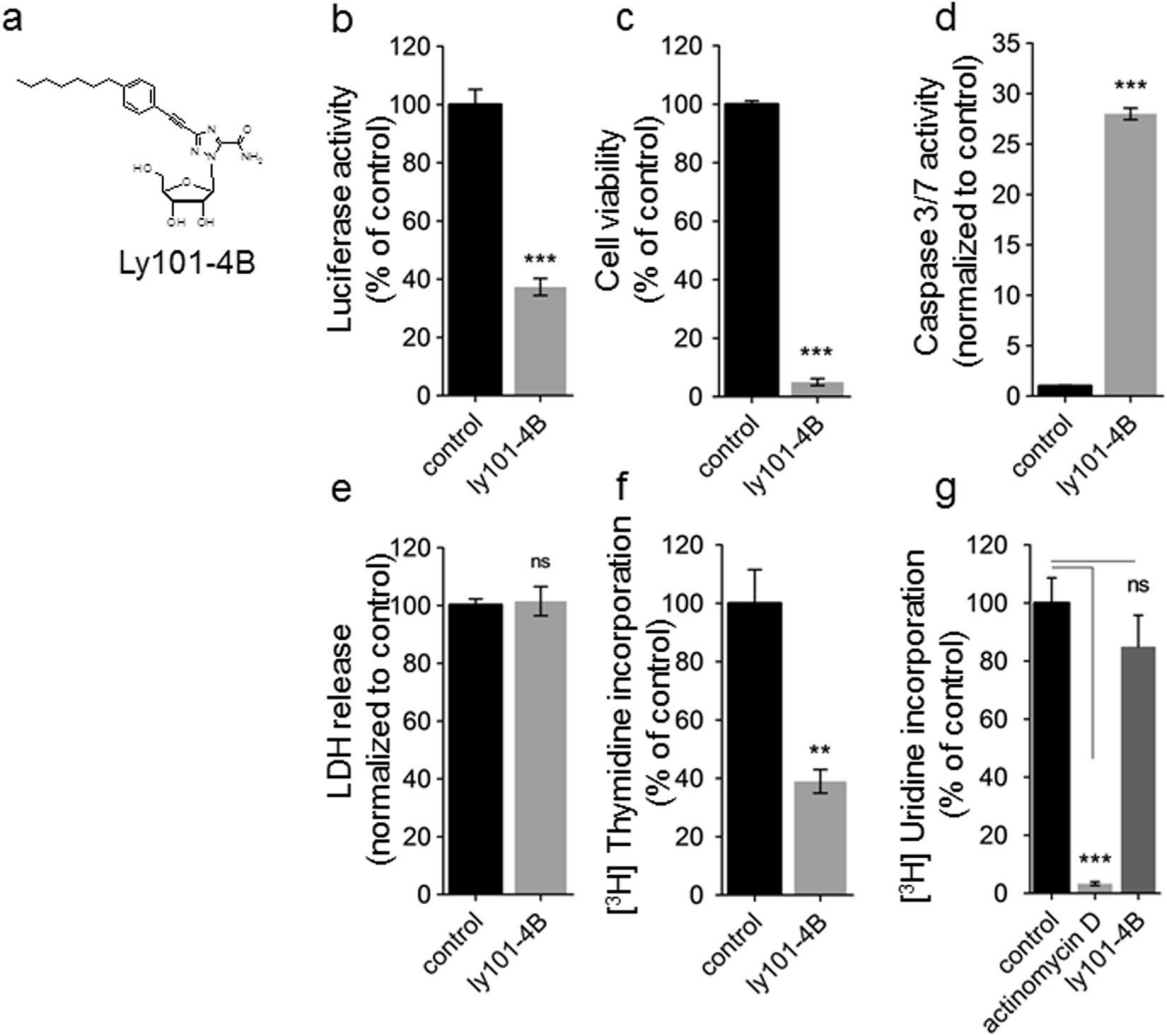
Characterization of the ly101-4B, a compound with an anti-E2F activity. (**a**) Molecular structure of the ly101-4B a triazole nucleoside compound. (**b**) Luciferase activity assay using the 6x E2F-luciferase reporter in MiaPaCa2 cell line treated during 72 h with ly101-4b (25 µM) or vehicle treated in control. (**c**) MiaPaca2 cell lines were treated as in b. Cell viability was assessed using the PrestoBlue reagent for 3 h incubation at 37 °C according to the manufacturer. Fluorescences values were blank substracted and normalized to control (vehicle treated) (**d**) Effects of ly101-4B on caspases 3 and 7 proteolytic activities. MiaPaca2 cells were treated 72 h with ly101-4b (25 µM). Results are expressed relative to control values (=1). Data are mean ± SEM of tree independent experiments. (**e**) Effect of ly101-4B on cytosolic LDH release. MiaPaca2 cells were treated 48 h with ly101-4B. Results are expressed as percentage relative to vehicle treated. Data are mean ± SEM of three independent experiments of different cultures, each one performed in triplicates. (**f**,**g**) MiaPaCa2 cells in exponential growth phase were treated with ly101-4B for 12 h and then labeled with [^3^H] Thymidine (10 μCi/ml) in f or [^3^H] Uridine (10 μCi/ml) in g for a 6 h period. In f, treatment with actinomycin d was used as a negative control of RNA synthesis. DNA and RNA synthesis rates were determined according to the radioactivity by using liquid scintillation counting. Each experiment was performed in triplicate. Data are expressed in percentage relative to vehicle treated condition.

Using a DNA construct containing a 6x E2F response element driving the luciferase reporter transfected on MiaPaCa2 cells, we screened a set of 176 original compounds with anticancer activity available at our laboratory^27–33^. Among them, 175 showed almost no effect on the reporter activity of the construct at the concentration which were used (data not shown). However, from these compounds we identified the nucleoside analogue ly101-4B (Fig. 4a) as a strong inhibitor of the E2F activity since the luciferase activity was reduced to 37.3 ± 2.9% (n = 3) of the control cells (Fig. 4b). We confirmed its activity by studying its effect on cell viability (Fig. 4c), caspase 3/7 activity (Fig. 4d) and LDH release (Fig. 4e) to measure cell growth, apoptosis and necrosis respectively in MiaPaCa2 cells after treatment with 25 µM. Cell viability was strongly decreased to 5.0 ± 1.1% (n = 3) of the control after 72 h of treatment. Caspase 3/7 activity was increased 28.0 ± 0.6 folds (n = 3) compared to control, whereas LDH release did not change, as presented in Fig. 4c–e. We also estimated DNA and RNA synthesis by measuring the [^3^H] Thymidine and [^3^H] Uridine incorporation respectively. DNA synthesis was decreased to 39.0 ± 4.0% (n = 3) of the control after 24 h of treatment whereas RNA synthesis remained unchanged as showed in Fig. 4f,g.

Then, in order to identify the pathways affected in MiaPaCa2 cells after the treatment by 24 h with ly101-4B and to confirm that E2F pathway activation is reduced upon treatment, we performed a transcriptome analysis by using an Affymetrix approach. As a first analysis we studied the expression of the E2F-dependent genes. We found that 151 of the 196 analyzed above were downregulated in treated cells with a p-value of >0.05 as presented in Supplementary Table 5. Then, the differentially expressed gene list was loaded into Ingenuity Pathway Analysis (IPA) 5.0 (http://www.ingenuity.com) and STRING^34^ bioinformatics tools to perform biological network and functional analyses. A total of 35 pathways associated to mitosis, cell cycle, DNA synthesis, nuclear division, G1/S transition were strongly inhibited by the treatment with ly101-4B as showed in Table 2. The FDR corresponding to these pathways was from 1.46e-59 to 6.44e-15.

**Table 2:** ly101-4B treated cells.

Pathway ID	Downregulated biological process	Observed genes	FDR
GO.0000278	mitotic cell cycle	73	1.46e-59
GO.0007049	cell cycle	80	4.09e-54
GO.0022402	cell cycle process	72	1.01e-51
GO.1903047	mitotic cell cycle process	64	8.23e-51
GO.0006259	DNA metabolic process	54	3.38e-39
GO.0051276	chromosome organization	57	1.03e-37
GO.0051301	cell division	41	3.16e-30
GO.0044770	cell cycle phase transition	36	4.36e-30
GO.0022616	DNA strand elongation	19	1.92e-29
GO.0006260	DNA replication	30	4.43e-28
GO.0006271	DNA strand elongation involved in DNA replication	18	6.24e-28
GO.0007067	mitotic nuclear division	35	7.08e-28
GO.0044772	mitotic cell cycle phase transition	34	8.98e-28
GO.0006281	DNA repair	36	4.7e-26
GO.0006996	organelle organization	75	4.7e-26
GO.0000280	nuclear division	36	6.25e-26
GO.0006974	cellular response to DNA damage stimulus	42	1.52e-25
GO.0090304	nucleic acid metabolic process	80	1.77e-20
GO.0006310	DNA recombination	24	2.16e-20
GO.1902589	single-organism organelle organization	56	2.2e-19
GO.0006261	DNA-dependent DNA replication	18	4.59e-19
GO.0033554	cellular response to stress	51	5.4e-19
GO.0006139	nucleobase-containing compound metabolic process	82	6.98e-19
GO.0046483	heterocycle metabolic process	83	1.97e-18
GO.0000819	sister chromatid segregation	17	5.17e-18
GO.1901360	organic cyclic compound metabolic process	84	8.65e-18
GO.0071840	cellular component organization or biogenesis	83	1.18e-17
GO.0051726	regulation of cell cycle	39	1.19e-17
GO.0016043	cellular component organization	81	5.1e-17
GO.0000070	mitotic sister chromatid segregation	15	1.17e-15
GO.0034641	cellular nitrogen compound metabolic process	83	1.2e-15
GO.0007059	chromosome segregation	20	1.58e-15
GO.0033260	nuclear DNA replication	11	3.39e-15
GO.0098813	nuclear chromosome segregation	17	4.42e-15
GO.0000082	G1/S transition of mitotic cell cycle	19	6.44e-15

### PDAC with activated E2F pathway are more sensitive to the ly101-4B treatment

**Figure 5:**
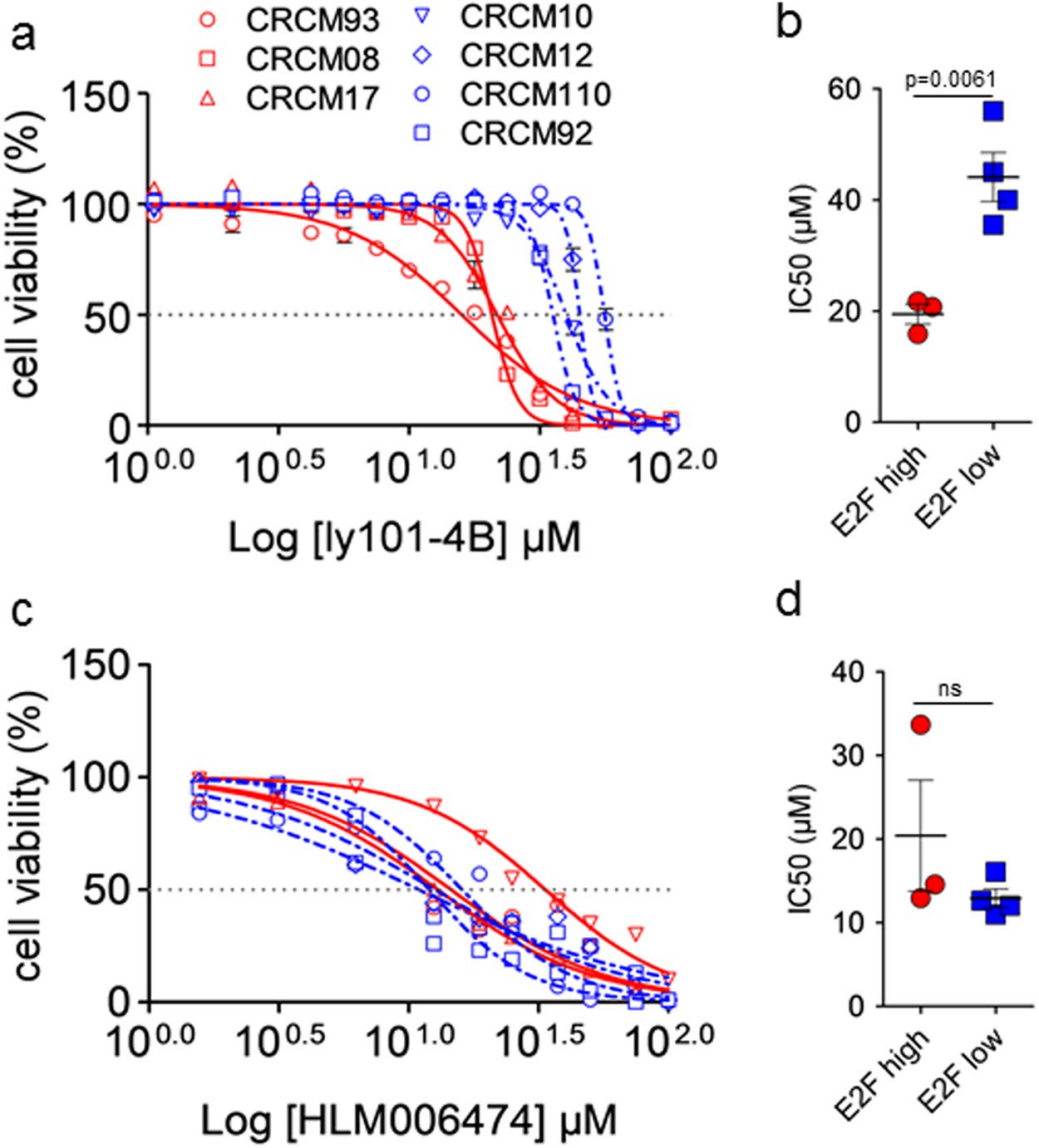
Effects of ly101-4B and HLM006474 on E2F high and low activity PDAC derived cell lines. (**a**,**b**) Three E2F high activity cell lines (in red) and four E2F low activity cell lines (in blue) were tested for their viability upon a 72 h treatment with both drugs. Cell viability was measured using the PrestoBlue reagent according to the manufacturer. Data are expressed as percentage relative to vehicle treated conditions in at least two independents experiments made in triplicates. (**b**,**d**) IC50 values were calculated by non-linear regression curves with robust fit using GraphPad software. Data are mean ± SEM.

We selected 3 primary cell cultures derived from PDX with high E2F targets level (CRCM93, CRCM08 and CRCM17) and 4 from PDX with low E2F targets level (CRCM10, CRCM12, CRCM110 and CRCM92) according to the normalized ratios (Fig. 2c). These cells were treated with increasing amounts of ly101-4B compound and after 72 h of treatment their viability was measured. Interestingly, all cells having the E2F highly activated resulted more sensitive to this treatment as showed in Fig. 5a. Figure 5b shows that the window of sensitivity (IC50) of the E2F high (19.4 ± 1.8 µM; n = 3) and E2F low (44.1 ± 4.4 µM; n = 4) cells. These cells were also treated with different concentrations (from 0 to 100 µM) of HLM006474, an inhibitor of E2F4 with anticancer activity identified by using a computer-based virtual screen^35^, for 72 h (Fig. 5c). We observed that although this compound is able to kill PDAC-derived cells, isn’t unable to discriminate between cells having E2F high (20.4 ± 6.6 µM; n = 3) and E2F low (12.9 ± 1.1 µM; n = 4) activity as presented in Fig. 5d. This result suggest that HLM006474, although inhibits E2F4 activity, have probably other E2F-unrelated targets. Altogether, we can affirm that using a set of genes indicators of the E2F activation we can select a group of PDAC more sensitive to E2F inhibitors. This strategy can be used to select sensitive patients and to propose a more adapted treatment than a general and unspecific cytotoxic drug.

## Discussion

The main goal of this study was to define whether or not there are strategies of clinical relevance to select patients with a PDAC for individualized treatments. For the moment all approaches focused to select genetic mutations and treat the patients on these targets failed almost completely with only rare exceptions that work, but only transitorily. This may be explained by the fact that downstream pathways of the mutated genes have some complex regulators. Therefore, targeting genes because they are mutated in PDAC is not enough justified because it is not certain that their regulated intracellular pathway became activated as presumed, and more important, that cells became addicts to these pathways. PDAC have several hundred mutations combined in a variable proportion among them, although the most frequently founded are common to the majority of patients, and in addition, common to other types of solid tumors^8,9,36^. Because these frequent mutations, or their combinations, present in the PDAC aren’t associated to the efficiency of a treatment or to their clinical outcome, we hypothesized that the reasons of this heterogeneity are controlled at post-genetic level such as epigenetically or transcriptionally. Consequently, the therapeutic targets for treating these patients should be classified based on their phenotypic characteristics such as for example differential RNAs expression. In this work we prepared 55 PDX and, analyzing their transcriptome by a bioinformatics unsupervised approach, we found two clinically different groups of patients supporting the hypothesis that the PDAC outcome is depending of their phenotype. Analyzing the activated pathways in these clusters of patients we found that one of the most differentially activated in the more aggressive group is the E2F-dependent pathway, which is in fact not a surprise since E2F is controlling cell growth, DNA synthesis, senescence and apoptosis. Therefore, we speculate that E2F-dependant pathway may control aggressiveness of the PDAC. Then, we realized a supervised clustering by focusing on the E2F-dependent genes and found that those patients with high E2F targets expression level survived less and showed a shorter RFS than patients with low E2F targets level. These data suggest that E2F is a crucial factor controlling the clinical outcome of patients with a PDAC as it has been suggested for other tumors^37,38^. Then, we speculated that the level of expression of E2F targets genes would control the chemosensitivity to standard anticancer cytotoxic drugs. Therefore, we realized 36 chemograms with 5 common cytotoxic drugs used for treating patients with a PDAC and compared their sensitivity with their E2F targets signature but we found no correlation as showed in Fig. 3. This observation is indicating that E2F targets signature is not crucial for efficiency of the cytotoxic activity with these anticancer drugs.

We next speculated that PDAC with high E2F targets level should be more sensitive to the inhibitors of the E2F activity. Therefore, we screened 176 compounds with anticancer effect, available at our laboratory, using a 6x E2F response element driving the luciferase reporter as readout to select compounds able to inhibit the E2F activity. We found that ly101-4B, a nucleoside analogue, decreased considerably the reporter activity to 37% of the control after 12 h of treatment. Whether this effect is direct on E2F factor or indirect remain to be determined. Treatment of the MiaPaCa2 cells with ly101-4B showed a strong decrease in cell survival, with a decrease in DNA, but not RNA, synthesis and a strong increase in caspase 3/7 activity but not a LDH release. Then, to investigate the mechanism of action of ly101-4B we performed a transcriptomic analysis of the MiaPaCa2-treated cells and found that, first, 151 of the 196 E2F-dependent genes were significantly down-regulated and, second, that STRING and IPA bioinformatics analysis demonstrated that cell cycle, mitosis, cell division and DNA replication pathways were considerably repressed as indicated in Table 2. Altogether, these results strongly suggest that ly101-4B is efficiently targeting E2F. Then, to validate our hypothesis we chose 3 primary cultures derived from PDX with E2F high activity and 4 with low activity and treated these cells with increasing concentration of the ly101-4B and another previously reported inhibitor of the E2F4 named HLM006474. As observed, treatment with ly101-4B showed an IC50 of 19.4 ± 1.8 for the E2F high cells whereas an IC50 of 44.1 ± 4.4 for E2F low cells. However, to our surprise, HLM006474 treatment was not discriminant between E2F-high and E2F-low primary cells as showed in Fig. 5. Other anticancer effects, totally unrelated to the E2F activity, of the HLM006474 compound can explain its incapability to discriminate between E2F-high and E2F-low tumors. At concentration of more than several µM it is not a surprise to found some off target effects for anticancer compounds. For example, it was previously reported that ly101-4B reduced the expression of the HSF1 in ovarian cancer cells^39,40^ that could be considered as an additional, and probably independent to the E2F inhibition, anticancer effect.

In conclusion, in this work we describe an E2F targets expression-based classification that could be predictive for patient outcome, but more important, the sensitivity to the E2F inhibitors but not sensitivity to cytotoxic anticancer drugs. Finally, we can assume that phenotypic characterization, essentially by an RNA expression analysis, of the PDAC can help to predict their clinical outcome and to predict their response to some treatments when were rationally selected.

## Material and Methods

### PDAC samples and cell culture

Patients were included under the Paoli Calmettes Institute clinical trial NCT01692873 (https://clinicaltrials.gov/show/NCT01692873). Three expert clinical centers collaborated on this project after receiving ethics review board approval. Consent’s forms of informed patients were collected and registered in a central database. The tumor tissues used for xenograft development was deemed excess to that required for the patient’s diagnosis. All the samples were anonymized. Two types of samples were obtained, namely Endoscopic Ultrasound-Guided Fine-Needle Aspiration (EUS-FNA) biopsies from patients with unresecable tumors, and tumor tissues from patients undergoing surgery. Fifty five xenografts were included. PDAC samples were mixed with 100 µl of Matrigel (BD Biosciences) and implanted with a trocar (10 Gauge, Innovative Research of America, Sarasota, FL) in the subcutaneous right upper flank of an anesthetized and disinfected mouse. When tumors reached 1 cm^3^, mice were sacrificed and tumors were removed.

To obtain primary cell cultures of these tumors, xenografts were splited into several small pieces and processed in a biosafety chamber: after a fine mincing, they were treated with collagenase type V (ref C9263; Sigma) and trypsin/EDTA (ref 25200-056; Gibco, Life Technologies) and suspended in DMEM supplemented with 1% w/w Penicillin/Streptomycin (Gibco, Life Technologies) and 10% Fetal Bovine Serum (Lonza). After centrifugation, cells were re-suspended in Serum Free Ductal Media (SFDM) adapted from Schreiber *et al*.^41^ without antibiotic and incubated at 37 °C in a 5% CO_2_ incubator.

### Chemograms

Cells were screened for chemosensitivity to five clinically used drugs in patients with PDAC: gemcitabine, 5-fluorouracil (5-FU), oxaliplatin, docetaxel and irinotecan. These cells were treated for 72 h with increasing concentrations of chemotherapeutic drugs ranging from 0 to 1000 µM. Five thousand cells were plated per well in 96-well plates in serum free defined media. Twenty-four hours later, the media were supplemented with increasing concentrations of drugs and were incubated for an additional 72 h period. Each experiment was performed in triplicate and repeated at least three times. Cell viability was estimated after addition of the PrestoBlue cell viability reagent (Life Technologies) for 3 h following the protocol provided by the supplier.

Three primary cell cultures with E2F high activity (CRCM93, CRCM08 and CRCM17) and 4 with E2F low activity (CRCM10, CRCM12, CRCM110 and CRCM92) were screened for their chemosensitivity to ly101-4B and HLM006474 (Sigma-Aldrich, France). Five thousand cells per well were plated in 96-wells plates in SFDM medium. Twenty four hours later the media was supplemented with increasing concentrations of ly101-4B or HLM006474 ranging from 0 to 100 µM and incubated for an additional 72 h period. Each experiment was done in triplicate and repeated at least two times.

### Gene expression microarrays

Total RNA was purified from xenografts using TRIzol® Reagent (Gibco, Life Technologies) according to the manufacturer. RNA Integrity Number (RIN) was calculated using the Agilent 2100 Bioanalyzer (Agilent Technologies, Santa Clara, CA). RNA samples that reached a RIN over 8 were used for microarray hybridization (GeneChip; Affymetrix Inc., Santa Clara, CA). The Genechip® Human Gene 2.0 ST Arrays were washed and stained using the Affymetrix GeneChip fluidic station 450 (protocol EukGE‐WS2v5_450) and were scanned using a GeneChip scanner 3000G7 (Affymetrix Inc., Santa Clara, CA). GeneChip operating software version 1.4 (Affymetrix Inc., Santa Clara, CA) was used to obtain chip images and for quality control. Microarray analysis was performed in duplicate for each PDX samples by the CHU de Québec Research Center Gene Expression Platform (Quebec City, Quebec, Canada). For each sample, the mean of duplicates is used for further computational analysis. Hierarchical clustering of complete linkage with the Euclidian metric (for samples) and Pearson correlation (for genes) was performed by the use of BRB-array tools version 4.6.0 (http://linus.nci.nih.gov/BRB-ArrayTools.html). The robustness (R) indices and the discrepancy (D) indices (BRB-array tools) were calculated to give an indication concerning the reproducibility of the clusters.

Effect of ly101-4B on the transcriptome of MiaPaCa2 cells was analyzed after the treatment with 25 µM for 24 h on the Affymetrix platform as described above.

The Gene Expression Omnibus (GEO) (http://www.ncbi.nlm.nih.gov/geo) accession numbers corresponding to our dataset are GSE55513 (17 samples) and GSE89792 (38 samples).

### Bioinformatics analysis

First of all, a pairewise-patients correlation analysis was performed to assess the high reproductibility of duplicate hybridization. Further, the expression level of probes was averaged between duplicates. All following statistical approaches were performed on that averaged expression value.

In study, we aimed to assess if transcriptomic subtypes could be associated with patient outcome. For this purpose, we described the natural clustering of patients on the basis of their transcriptomic profiles using the agglomerative hierarchical clustering (AHC) method with Euclidean distance and complete linkage^42^. To assess the robustness of our clustering pattern, we used a reclustering method with data disruption by introducing Gaussian background noise as described in Zhao *et al*.^43^. Two indices of the clustering robustness are provided. The R index measures the reproducibility of the cluster. An index R of 1 means perfect reproducibility. The D index measures the number of discrepancies (additions or omissions) comparing an original cluster to a cluster that best matches the perturbed data. An index R of 0 no discrepancies despite data perturbation. The heatmap illustrations were made on GENE‐E software (version 3.0.204; Broad Institute, Cambridge, MA, USA). Differentially expressed genes between the AHC clusters were identified using permutation t‐test with welch correction. The pvalues were adjusted for false discovery rate using Benjamini & Hochberg method^44^. The threshold of significance was set at adjusted-p-value < 0.05. Gene set enrichment analysis (GSEA) was performed using the Broad Institute platform and statistical significance (false discovery rate) was seated at 0.05.

### Screening of compounds with E2F inhibition ability

A library containing 176 triazole nucleoside compounds with anticancer activity was previously developed^27–33^ and used to screen for detecting compounds with an inhibitory activity on the E2F transcription factor. Transactivation assays were performed on MiaPaCa2 cells transiently transfected with 1 µg of 6x E2F-luciferase reporter, plus 0.5 µg of the pCMV-β-gal for normalization, and treated for 12 h with 1 and 10 µM of each compound. The % of inhibition of the E2F reporter was calculated in the presence or absence of the compound. Experiments were performed in triplicate.

### Cell viability, caspase 3/7 activity, LDH release, [^3^H] Thymidine and [^3^H] Uridine incorporation

MiaPaCa2 cells were initially seeded at 5000 cells/well on 96-well plates. After 24 h cells were treated with 25 µM of ly101-4B and cell viability was estimated 72 h later by the addition of PrestoBlue™ reagent (Life Technologies) for 3 h following the PrestoBlue™ cell viability reagent protocol provided by the supplier. Caspase-3/7 activity was measured by using the Apo-ONE homogeneous caspase-3/7 assay fluorometric kit (Promega). Cells were treated with the compound for 48 h and caspase-3 activity was measured by the cleavage of the fluorometric substrate Z-DEVD-R110 according to the instructions of the manufacturer (Promega). The LDH release after 48 h of treatment with ly101-4B was measured by using a commercial LDH kit (cytotoxicity detection kit, Roche) according to the manufacturer’s protocol (Roche Diagnostics). MiaPaCa2 cells in exponential growth phase were treated with ly101-4B for 12 h and then labeled with [^3^H] Thymidine (10 μCi/ml) or [^3^H] Uridine (10 μCi/ml) for a 6 h period. Treatment with actinomycin D was used as a control of RNA synthesis. Then the cells were harvested and DNA and RNA synthesis activity was determined according to the radioactivity by using liquid scintillation counting. Each experiment was performed in triplicate.

### Statistical analysis

The overall survival and relapse‐free survival were analyzed using the Kaplan-Meier log‐rank test to assess differences in survival. A t-test was used to assess the statistical significance of mean differences between two groups.

The IC50 values were calculated from a log ([drug]) versus normalized response curve with robust fit using GraphPad Prism software v5.0 (GraphPad Software). Data for cell viability assays were analyzed using one-way repeated analysis of variance (ANOVA) with Dunnett post-hoc test for multiple comparisons. A p-value < 0.05 is considered significative.

## Electronic supplementary material

Supplementary data
