# Supplementary material for: E2F signature is predictive for the pancreatic adenocarcinoma clinical outcome and sensitivity to E2F inhibitors, but not for the response to cytotoxic-based treatments
Source: Sci Rep. 2018 May 29;8:8330. doi: 10.1038/s41598-018-26613-z (PMC5974374; doi:10.1038/s41598-018-26613-z)
Supplement: Supplementary file 1 — Supplementary data [file 41598_2018_26613_MOESM1_ESM.pdf]

E2F signature is predictive for the pancreatic adenocarcinoma clinical outcome and sensitivity to E2F inhibitors, but not for the response to cytotoxic-based treatments

Wenjun Lan[1,2,4], Benjamin Bian[1,4], Yi Xia[3], Samir Dou[1], Odile Gayet[1], Martin Bigonnet[1], Patricia Santofimia-Castaño[1], Mei Cong[2], Ling Peng[2], Nelson Dusetti[1], Juan Iovanna[1]

[1]Centre de Recherche en Cancérologie de Marseille (CRCM), INSERM U1068, CNRS UMR 7258, Aix-Marseille Université and Institut Paoli-Calmettes, Parc Scientifique et Technologique de Luminy, Marseille, France.

[2]Aix-Marseille Université, CNRS, Centre Interdisciplinaire de Nanoscience de Marseille, UMR 7325, «Equipe Labellisée Ligue Contre le Cancer», Marseille, France.

[3]Chongqing Key Laboratory of Natural Product Synthesis and Drug Research, School of Pharmaceutical Sciences, Chongqing University, Chongqing, China.

[4]Equal contribution

Supplementary Information

Supplementary Figure 1

IPA network analysis of the top-ranked uptream regulators in the E2F-high subgroup. Upstream regulator analysis identifies the cascade of upstream transcriptional regulators that can explain the observed gene expression changes in our dataset. Activated (z-score ≥ 2) and inhibited (z-score ≤ −2) upstream regulators are highlighted in orange and blue, respectively. Up-regulated and down-regulated genes are highlighted in red and green, respectively, and the color depth is correlated to the fold change. Orange and blue dashed lines with arrows indicate indirect activation and inhibition, respectively. Yellow and gray dashed lines with arrows depict inconsistent effects and no prediction, respectively.

Supplementary Figure 2

E2F's isoforms expression in the both E2F subgroup of PDX. Each E2F's isoform were screened for their expression in both PDX subgroups. The Affymetrix Hugene ST 2.0 array probeset ID is given for each isoform. The boxplots represent distribution of RMA normalized data. The line in the box-plot representation shows the median value of mRNA expression ratios, the lower and upper limits of each box represents the first and third quartiles, respectively. Whiskers represent the limits of extreme measurements. p-values are given according to the two-tailed non parametric Mann and Whitney t-test with Gaussian approximation.

Supplementary Figure 3

External cohort validation of the 24-genes E2F signature: **A**: RNA-sequencing data were selected from TCGA-PAAD cohort of 178 patients with PDAC. The hierarchical clustering of the 178 samples was made according to the expression of the 21-genes signature. Euclidian distance metric with complete linkage was applied to this cohort as the same metric applied to the learning cohort of 55 PDX samples. Two clusters of samples named respectively TCGA E2F-high (n=17) and TCGA E2F-low (n=161) were characterized for their disease free and overall survival (**B**). Kaplan-Meier curves show that the median of DFS is significantly reduced in the TCGA E2F-high subgroup (8.2 months) compared to TCGA E2F low subgroup (22.5 months). p-values were compute according to Log-rank (Mantel-Cox) Test.

Supplementary Figure 4

Heatmap showing the selected markers. Eleven up-regulated genes (upper panel) and thirteen down-regulated genes (bottom panel) were selected according to their raw p-value (<0.002), FDR p-value (<0.05), t-test score (>5 and ≤-5) and their fold change (>1.5 and ≤ -1.5).

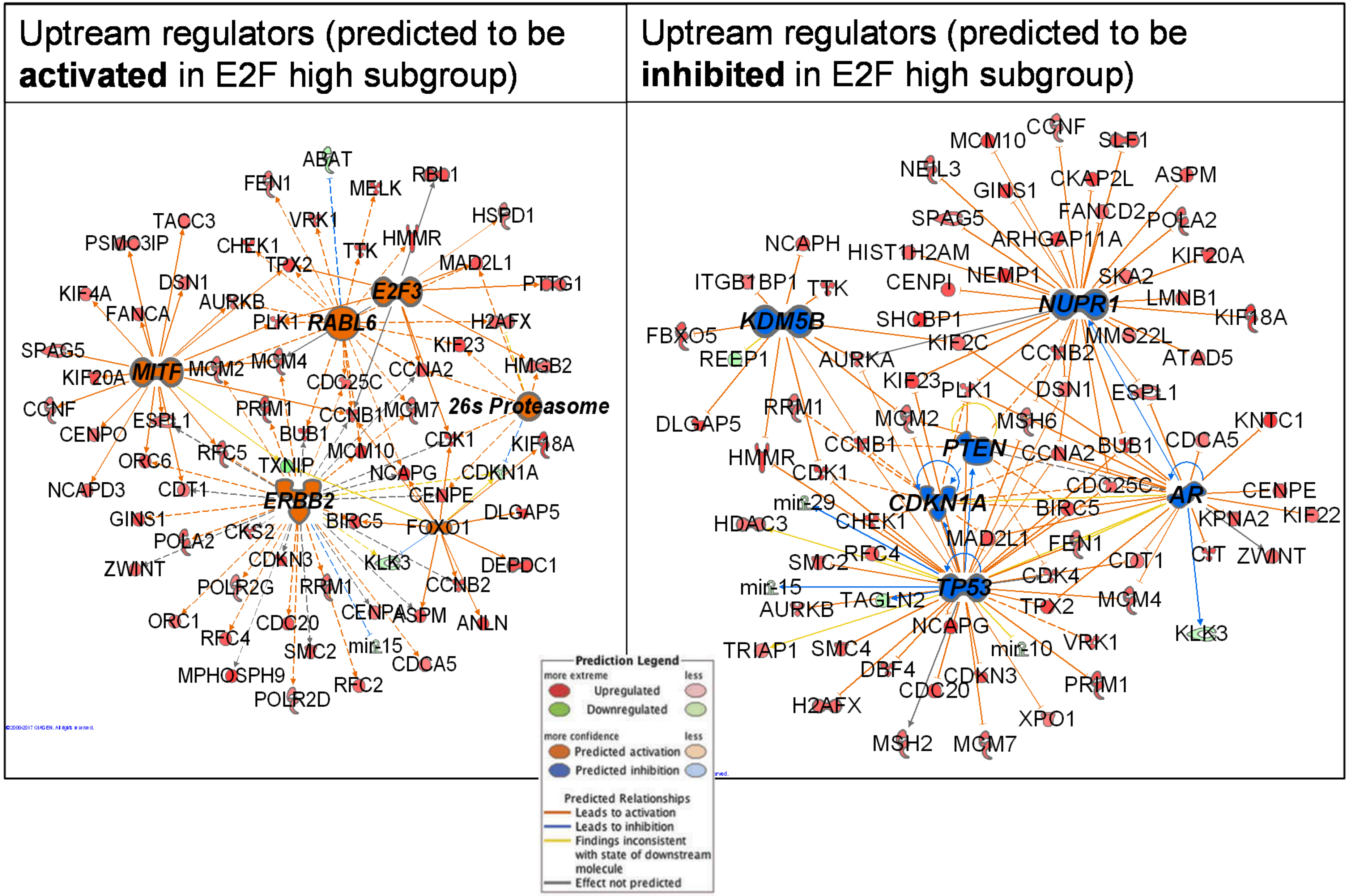

Supplementary Figure 1

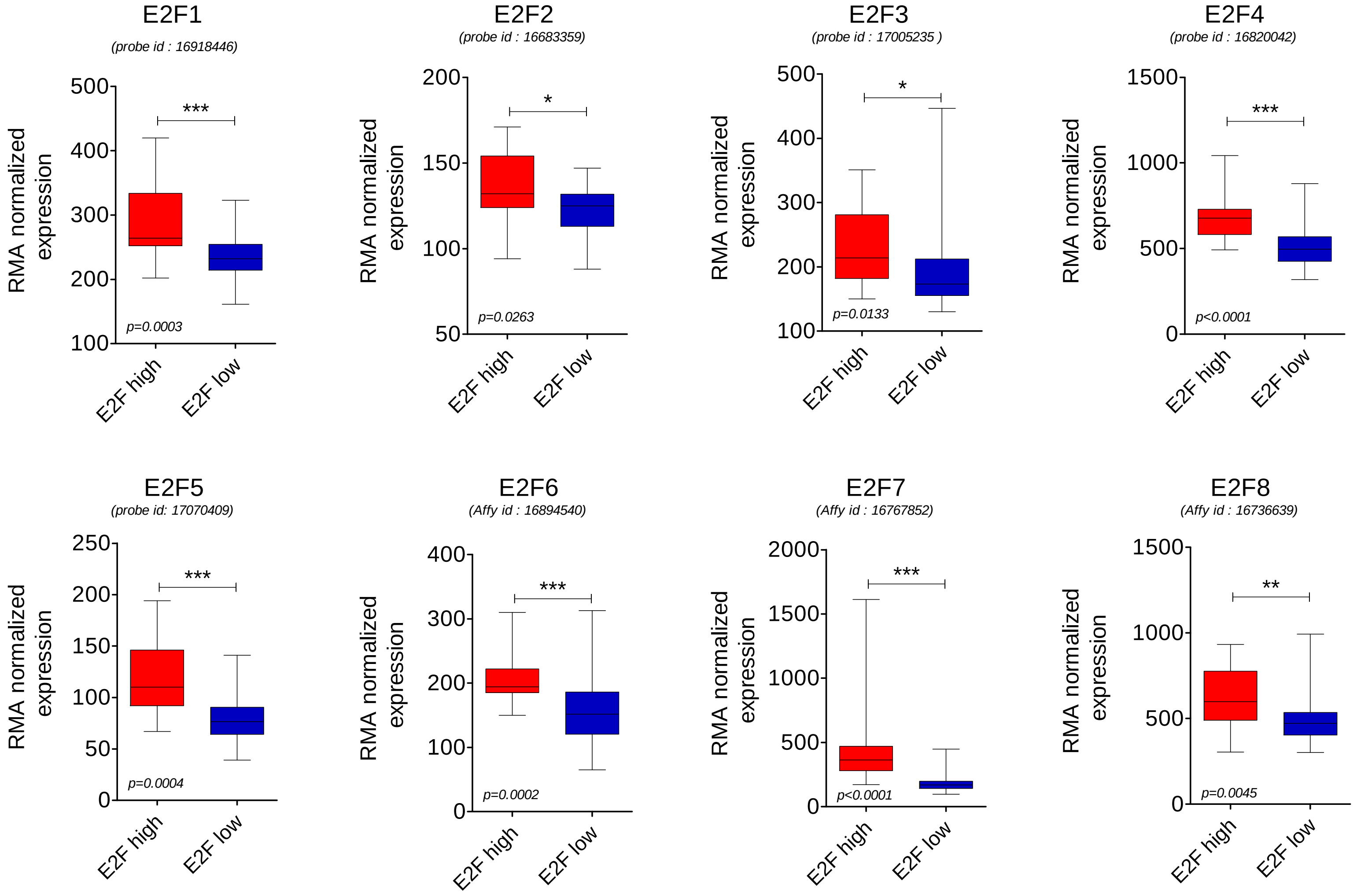

Supplementary Figure 2

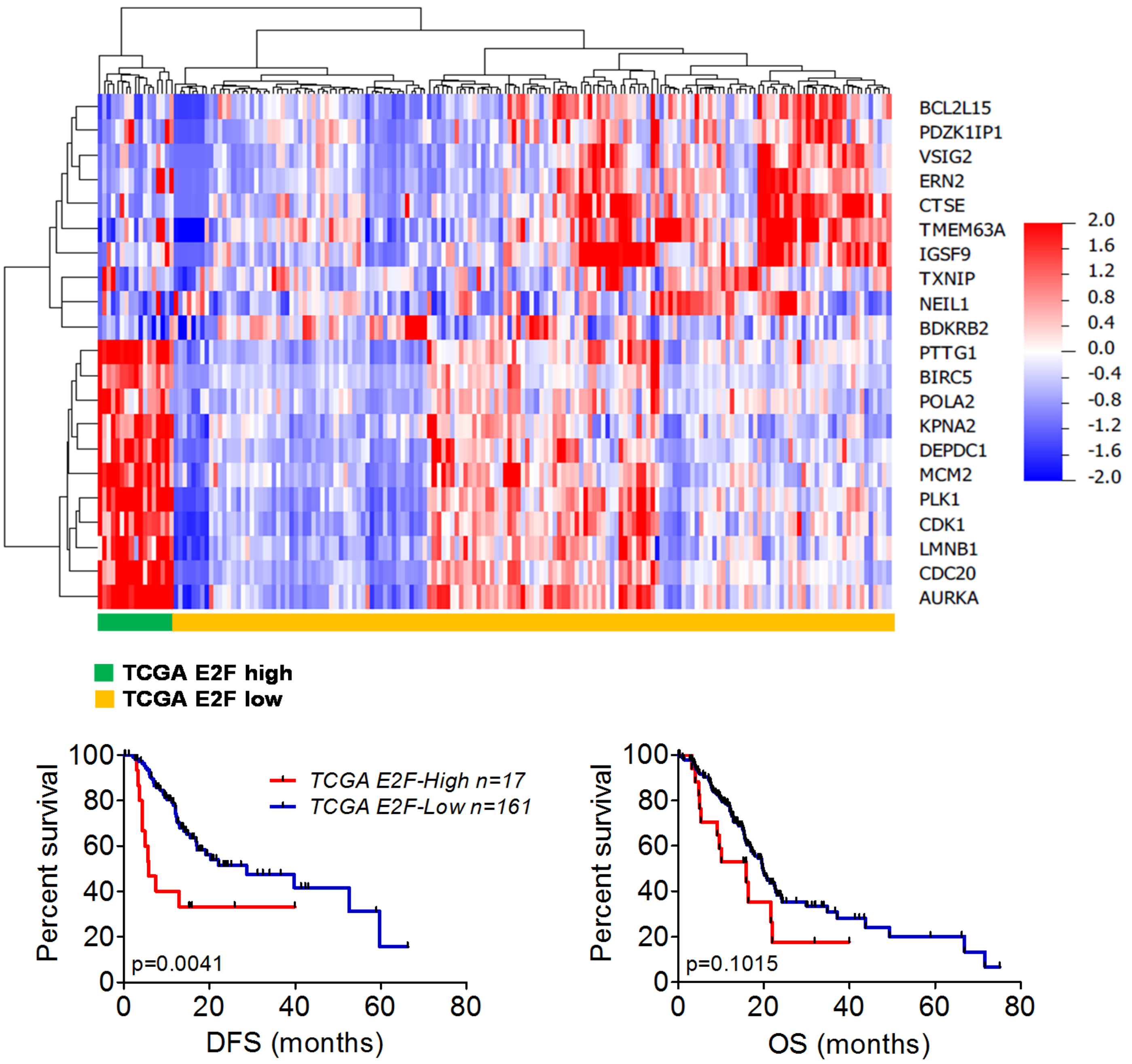

Supplementary Figure 3

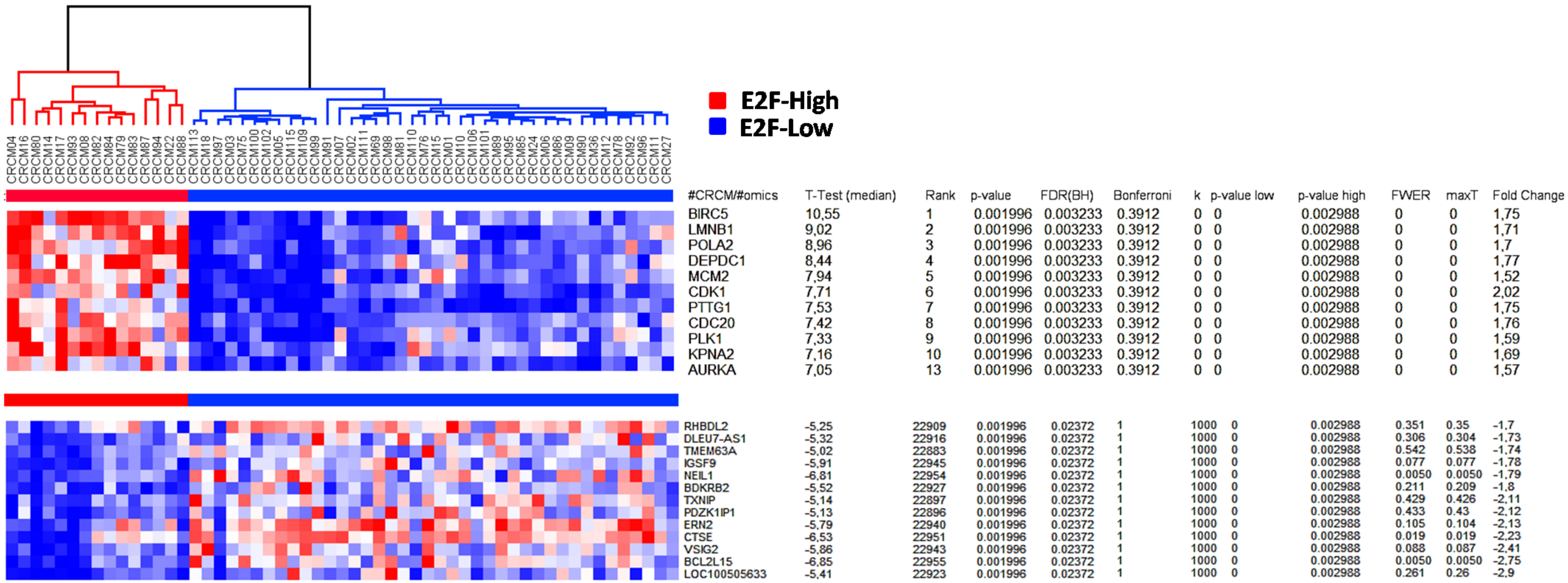

#CRCM/#omics	T-Test (median)	Rank	p-value	FDR(BH)	Bonferroni	k	p-value low	p-value high	FWER	maxT	Fold Change
BIRC5	10,55	1	0.001996	0.003233	0.3912	0	0	0.002988	0	0	1,75
LMNB1	9,02	2	0.001996	0.003233	0.3912	0	0	0.002988	0	0	1,71
POLA2	8,96	3	0.001996	0.003233	0.3912	0	0	0.002988	0	0	1,7
DEPDC1	8,44	4	0.001996	0.003233	0.3912	0	0	0.002988	0	0	1,77
MCM2	7,94	5	0.001996	0.003233	0.3912	0	0	0.002988	0	0	1,52
CDK1	7,71	6	0.001996	0.003233	0.3912	0	0	0.002988	0	0	2,02
PTTG1	7,53	7	0.001996	0.003233	0.3912	0	0	0.002988	0	0	1,75
CDC20	7,42	8	0.001996	0.003233	0.3912	0	0	0.002988	0	0	1,76
PLK1	7,33	9	0.001996	0.003233	0.3912	0	0	0.002988	0	0	1,59
KPNA2	7,16	10	0.001996	0.003233	0.3912	0	0	0.002988	0	0	1,69
AURKA	7,05	13	0.001996	0.003233	0.3912	0	0	0.002988	0	0	1,57

RHBDL2	-5,25	22909	0.001996	0.02372	1	1000	0	0.002988	0.351	0.35	-1,7
DLEU7-AS1	-5,32	22916	0.001996	0.02372	1	1000	0	0.002988	0.306	0.304	-1,73
TMEM63A	-5,02	22883	0.001996	0.02372	1	1000	0	0.002988	0.542	0.538	-1,74
IGSF9	-5,91	22945	0.001996	0.02372	1	1000	0	0.002988	0.077	0.077	-1,78
NEIL1	-6,81	22954	0.001996	0.02372	1	1000	0	0.002988	0.0050	0.0050	-1,79
BDKRB2	-5,52	22927	0.001996	0.02372	1	1000	0	0.002988	0.211	0.209	-1,8
TXNIP	-5,14	22897	0.001996	0.02372	1	1000	0	0.002988	0.429	0.426	-2,11
PDZK1IP1	-5,13	22896	0.001996	0.02372	1	1000	0	0.002988	0.433	0.43	-2,12
ERN2	-5,79	22940	0.001996	0.02372	1	1000	0	0.002988	0.105	0.104	-2,13
CTSE	-6,53	22951	0.001996	0.02372	1	1000	0	0.002988	0.019	0.019	-2,23
VSIG2	-5,86	22943	0.001996	0.02372	1	1000	0	0.002988	0.088	0.087	-2,41
BCL2L15	-6,85	22955	0.001996	0.02372	1	1000	0	0.002988	0.0050	0.0050	-2,75
LOC100505633	-5,41	22923	0.001996	0.02372	1	1000	0	0.002988	0.261	0.26	-2,9

Supplementary Figure 4

id	CRCM80	CRCM80r	CRCM14	CRCM14r	CRCM08	CRCM08r	CRCM04	CRCM04r	CRCM17	CRCM17r	CRCM16	CRCM16r	CRCM91	[illegible]
CRCM80	0	6337,18	27977,78	28187,81	30153,55	30816,08	37415,51	37745,34	37104,11	37413,79	32208,08	32207,27	41747,35	[illegible]
CRCM80r	6337,18	0	27934,02	28079,69	29803,33	30477,74	37301,02	37768,32	37086,83	37305,12	32239,94	32300,46	41739,78	[illegible]
CRCM14	27977,78	27934,02	0	7620,94	31673,42	32739,47	34119,64	34414,13	35711,73	36068,1	35086,7	35157,84	42309,05	[illegible]
CRCM14r	28187,81	28079,69	7620,94	0	31308,2	32245,42	33433,06	34037,97	35069,52	35027	34869,47	34974,38	42061,63	[illegible]
CRCM08	30153,55	29803,33	31673,42	31308,2	0	5979,75	31888,81	32501,61	30517,18	30560,45	31885,31	31932,62	42665,07	[illegible]
CRCM08r	30816,08	30477,74	32739,47	32245,42	5979,75	0	31906,19	32601,8	30648,34	30638,68	32328,71	32347,1	43452,55	[illegible]
CRCM04	37415,51	37301,02	34119,64	33433,06	31888,81	31906,19	0	5600,54	28464,3	28510,97	29470,76	29564,36	46197,33	[illegible]
CRCM04r	37745,34	37768,32	34414,13	34037,97	32501,61	32601,8	5600,54	0	28806,96	29066,9	29528,49	29581,68	46467,75	[illegible]
CRCM17	37104,11	37086,83	35711,73	35069,52	30517,18	30648,34	28464,3	28806,96	0	6388,65	30879,59	30958,1	44751,92	[illegible]
CRCM17r	37413,79	37305,12	36068,1	35027	30560,45	30638,68	28510,97	29066,9	6388,65	0	31586,81	31721,73	44963,23	[illegible]
CRCM16	32208,08	32239,94	35086,7	34869,47	31885,31	32328,71	29470,76	29528,49	30879,59	31586,81	0	4817,04	40902,43	[illegible]
CRCM16r	32207,27	32300,46	35157,84	34974,38	31932,62	32347,1	29564,36	29581,68	30958,1	31721,73	4817,04	0	41078,03	[illegible]
CRCM91	41747,35	41739,78	42309,05	42061,63	42665,07	43452,55	46197,33	46467,75	44751,92	44963,23	40902,43	41078,03	0	[illegible]
CRCM91r	41202,82	41085,88	41715,8	41356,02	42073,29	42851,09	45649,69	45993,1	44226,08	44382,24	40332,67	40498,96	4971,72	[illegible]
CRCM76	35582,6	35509,6	39250,91	39349,4	34798,02	35430,39	41895,49	42030,09	40689,94	41254	33789,45	33779,83	38060,19	[illegible]
CRCM76r	34960,08	34576,49	38360,63	38388,02	34358,13	35134,23	41230,91	41504,31	40251,04	40665,51	33316,38	33397,62	37640,35	[illegible]
CRCM12	29567,97	29001,87	35124,2	34845,02	29649,73	30193,89	34588,68	35074,97	33555,6	34119,38	30400,68	30397,13	36311,05	[illegible]
CRCM12r	29816,07	29473,48	34639,7	34476,29	30937,96	32052,5	35044,7	35340,64	34167,1	34686,48	30422,04	30503,85	35671,55	[illegible]
CRCM83	39982,94	40138,74	43504,76	43414,53	39969,41	40357,03	47136,09	47348,86	45697,65	45939,95	39897,97	39922,16	41618,82	[illegible]
CRCM83r	40499,33	40665,23	44045,67	44082,16	40478,88	40917,72	47842,38	47996,54	46255,66	46653,64	40408,28	40442,02	42177,09	[illegible]
CRCM18	43969,89	42835,79	45449,69	45055,71	42547,86	43233,16	51224,73	51935,24	50870,88	51262,04	45305,64	45444,42	47750,03	[illegible]
CRCM18r	44657,29	43440,38	46084,36	45714	43446,93	44150,86	51903,72	52652,88	51558,61	52006,64	46155,27	46312,95	48630,71	[illegible]
CRCM75	44344,89	43680,47	45561,7	45050,86	40985,84	41678,66	48733,54	49281,84	48119,55	48459,55	40473,26	40581,93	45688,3	[illegible]
CRCM75r	44001,71	43295,43	45286,79	44707,27	40604,69	41281,9	48415,06	49005,96	47792,41	48088,77	40163,14	40284,29	45351,19	[illegible]
CRCM113	49724,55	49239,2	52049,71	51926,97	47761,78	48346,2	58356,88	58776,7	56956,61	57187,5	48714,26	48711,52	52758,82	[illegible]
[illegible]	49161,42	48571,9	51533,65	51391,6	47185,41	47769,87	57757,45	58243,4	56381,69	56618,82	48224,38	48225,9	52402,81	[illegible]
CRCM36	43387,25	42950,54	45184,96	44744,66	41655,52	42189,6	50507,01	50952,21	49736,92	49947,53	40650,12	40677,84	46846,38	[illegible]
CRCM36r	43590,86	43056,89	45476,79	45094,68	41970,12	42554,34	50834,78	51307,94	50072,34	50342,18	41067,77	41098,66	47053,21	[illegible]
CRCM100	46663,01	46061,36	49424,98	49065,71	45699,62	46116,59	54120,53	54654,43	53241,62	53566,32	45782,93	45836,3	49993,7	[illegible]
[illegible]	46876,46	46188,68	49449,16	49032,41	45823,53	46258,61	54051,71	54631,84	53218,98	53500,7	45814,84	45893,33	50088,93	[illegible]
CRCM102	41569,46	40749,36	43199,38	42854,52	40658,08	41263,95	49393,97	50017,73	48630,74	48995,39	40674,76	40747,05	45945,86	[illegible]
[illegible]	41668,97	40864,44	43270,14	42968,72	40856,92	41442,58	49534,33	50147,43	48739,1	49174,82	40790,02	40880,86	46031,32	[illegible]
[illegible]	[illegible]	[illegible]	[illegible]	[illegible]	[illegible]	[illegible]	[illegible]	[illegible]	[illegible]	[illegible]	[illegible]	[illegible]	[illegible]	[illegible]

Supplementary Table 1

Supplementary Table 2

Gene name	T-Test (median)	p-value	FDR(BH)	Bonferroni	k	FWER	maxT	Fold Change
HINT1	9,75	0.001996	0.04489	1	0	0	0	1,55
PRMT7	8,41	0.001996	0.04489	1	0	0.0020	0.0020	1,26
FOXK2	8,26	0.001996	0.04489	1	0	0.0020	0.0020	1,34
TRMU	8,22	0.001996	0.04489	1	0	0.0020	0.0020	1,27
EIF3L	7,46	0.001996	0.04489	1	0	0.017	0.017	1,58
ICT1	7,21	0.001996	0.04489	1	0	0.031	0.031	1,6
NMRAL1	7,1	0.001996	0.04489	1	0	0.041	0.041	1,4
BCKDK	6,97	0.001996	0.04489	1	0	0.050	0.050	1,31
POLR3H	6,82	0.001996	0.04489	1	0	0.079	0.079	1,42
ZC3H12C	6,76	0.001996	0.04489	1	0	0.097	0.097	2,59
HSPA9	6,76	0.001996	0.04489	1	0	0.097	0.097	1,71
GGH	6,7	0.001996	0.04489	1	0	0.104	0.104	1,64
EME1	6,68	0.001996	0.04489	1	0	0.109	0.109	1,49
PFDN4	6,65	0.001996	0.04489	1	0	0.115	0.115	1,39
PPP2CA	6,59	0.001996	0.04489	1	0	0.138	0.138	1,36
C5orf24	6,52	0.001996	0.04489	1	0	0.155	0.155	1,5
XPNPEP3	6,52	0.001996	0.04489	1	0	0.155	0.155	1,48
HNRNPA0	6,5	0.001996	0.04489	1	0	0.159	0.159	1,42
CHAF1B	6,49	0.001996	0.04489	1	0	0.159	0.159	1,55
FTSJ3	6,35	0.001996	0.04489	1	0	0.215	0.215	1,27
MRPL38	6,24	0.001996	0.04489	1	0	0.259	0.258	1,27
IRAK2	6,19	0.001996	0.04489	1	0	0.283	0.282	1,67
HSPA4	6,19	0.001996	0.04489	1	0	0.287	0.286	1,68
GRPEL2	6,16	0.001996	0.04489	1	0	0.303	0.302	1,64
DDX46	6,14	0.001996	0.04489	1	0	0.312	0.311	1,43
RDX	6,14	0.001996	0.04489	1	0	0.312	0.311	2,17
NOL11	6,12	0.001996	0.04489	1	0	0.322	0.321	1,46
PSMC5	6,1	0.001996	0.04489	1	0	0.332	0.331	1,31
SLC9A7	6,08	0.001996	0.04489	1	0	0.342	0.341	1,55
EIF3D	6,07	0.001996	0.04489	1	0	0.351	0.35	1,38
CDKN2AIPNL	6,07	0.001996	0.04489	1	0	0.351	0.35	1,76
RAD50	6,06	0.001996	0.04489	1	0	0.364	0.363	1,6
SLC9A7	6,02	0.001996	0.04489	1	0	0.38	0.38	1,58
DRG1	6	0.001996	0.04489	1	0	0.397	0.397	1,58
HSPA9	6	0.001996	0.04489	1	0	0.399	0.399	2,03
C6orf132	-6,04	0.001996	0.04489	1	1000	0.369	0.369	-1,77
CYP2C18	-6,06	0.001996	0.04489	1	1000	0.364	0.363	-4
C6orf222	-6,07	0.001996	0.04489	1	1000	0.349	0.348	-4,14
CEACAM6	-6,08	0.001996	0.04489	1	1000	0.342	0.341	-3,43
AQP2	-6,09	0.001996	0.04489	1	1000	0.335	0.334	-1,22
FUT3	-6,1	0.001996	0.04489	1	1000	0.335	0.334	-2,64
MIR27B	-6,11	0.001996	0.04489	1	1000	0.328	0.327	-1,96
VILL	-6,13	0.001996	0.04489	1	1000	0.315	0.314	-2,52
OMP	-6,13	0.001996	0.04489	1	1000	0.315	0.314	-1,44
MBOAT1	-6,15	0.001996	0.04489	1	1000	0.306	0.305	-2,22
TLN2	-6,15	0.001996	0.04489	1	1000	0.305	0.304	-1,75
TNIK	-6,16	0.001996	0.04489	1	1000	0.301	0.30	-2,35
LINC00675	-6,17	0.001996	0.04489	1	1000	0.293	0.292	-3,09
TMEM133	-6,18	0.001996	0.04489	1	1000	0.289	0.288	-2,45
MIR192	-6,18	0.001996	0.04489	1	1000	0.289	0.288	-3,04
TLE4	-6,18	0.001996	0.04489	1	1000	0.288	0.287	-1,56
ABHD2	-6,23	0.001996	0.04489	1	1000	0.265	0.264	-1,96
FRK	-6,25	0.001996	0.04489	1	1000	0.252	0.251	-2,28
TMEM59	-6,25	0.001996	0.04489	1	1000	0.252	0.251	-1,4
ENST00000550175	-6,26	0.001996	0.04489	1	1000	0.244	0.243	-1,21
PLEKHG1	-6,27	0.001996	0.04489	1	1000	0.244	0.243	-2,11
BLNK	-6,27	0.001996	0.04489	1	1000	0.24	0.239	-2,52
VIL1	-6,29	0.001996	0.04489	1	1000	0.236	0.235	-3,36
EFCAB14	-6,32	0.001996	0.04489	1	1000	0.227	0.226	-1,38
RNF183	-6,32	0.001996	0.04489	1	1000	0.226	0.225	-1,62
BCAS1	-6,38	0.001996	0.04489	1	1000	0.205	0.205	-2,8
LGALS4	-6,41	0.001996	0.04489	1	1000	0.19	0.19	-3,47
FUT4	-6,44	0.001996	0.04489	1	1000	0.182	0.182	-1,88
PIK3C2B	-6,45	0.001996	0.04489	1	1000	0.177	0.177	-2,9
LOC100996583	-6,54	0.001996	0.04489	1	1000	0.151	0.151	-2,57
MYO1A	-6,57	0.001996	0.04489	1	1000	0.141	0.141	-4,16
PLA2G10	-6,59	0.001996	0.04489	1	1000	0.139	0.139	-3,73
SLC37A1	-6,64	0.001996	0.04489	1	1000	0.117	0.117	-1,92
ICA1	-6,66	0.001996	0.04489	1	1000	0.114	0.114	-2,03
LRRC31	-6,7	0.001996	0.04489	1	1000	0.104	0.104	-3,92
BC038570	-6,72	0.001996	0.04489	1	1000	0.101	0.101	-2,14
SDR16C5	-6,72	0.001996	0.04489	1	1000	0.099	0.099	-2,54
AMN	-6,72	0.001996	0.04489	1	1000	0.099	0.099	-1,73
SLC44A3	-6,72	0.001996	0.04489	1	1000	0.099	0.099	-2,33
SPDEF	-6,74	0.001996	0.04489	1	1000	0.098	0.098	-2,55
RALGPS1	-6,78	0.001996	0.04489	1	1000	0.086	0.086	-1,92

ABP1	-6,78	0.001996	0.04489	1	1000	0.086	0.086	-2,13
LOC100129473	-6,78	0.001996	0.04489	1	1000	0.086	0.086	-1,42
BCMO1	-6,8	0.001996	0.04489	1	1000	0.084	0.084	-2,41
TPMT	-6,82	0.001996	0.04489	1	1000	0.079	0.079	-2,22
FBP1	-6,83	0.001996	0.04489	1	1000	0.079	0.079	-2,75
MUC1	-6,83	0.001996	0.04489	1	1000	0.079	0.079	-4,43
TMEM51-AS1	-6,84	0.001996	0.04489	1	1000	0.075	0.075	-2,21
TLCD2	-6,85	0.001996	0.04489	1	1000	0.073	0.073	-1,78
TMEM125	-6,89	0.001996	0.04489	1	1000	0.065	0.065	-1,52
ATP8A1	-6,89	0.001996	0.04489	1	1000	0.065	0.065	-2,31
LDLR	-6,89	0.001996	0.04489	1	1000	0.065	0.065	-1,5
ELF3	-6,9	0.001996	0.04489	1	1000	0.063	0.063	-2,12
ST14	-6,9	0.001996	0.04489	1	1000	0.062	0.062	-2,56
PLCH1	-6,93	0.001996	0.04489	1	1000	0.057	0.057	-2,51
EPS8L3	-6,94	0.001996	0.04489	1	1000	0.056	0.056	-3,21
ATP2A3	-6,97	0.001996	0.04489	1	1000	0.054	0.054	-2,33
CXCL16	-6,99	0.001996	0.04489	1	1000	0.046	0.046	-2,56
CREB3L1	-7	0.001996	0.04489	1	1000	0.046	0.046	-2,62
IGSF9	-7,13	0.001996	0.04489	1	1000	0.039	0.039	-2,18
TXNIP	-7,15	0.001996	0.04489	1	1000	0.038	0.038	-2,96
EPHA10	-7,15	0.001996	0.04489	1	1000	0.038	0.038	-1,42
FOXP4	-7,18	0.001996	0.04489	1	1000	0.036	0.036	-1,48
TRIM2	-7,21	0.001996	0.04489	1	1000	0.031	0.031	-1,98
REG4	-7,21	0.001996	0.04489	1	1000	0.031	0.031	-12,03
SLC6A20	-7,25	0.001996	0.04489	1	1000	0.028	0.028	-3,6
MACC1	-7,31	0.001996	0.04489	1	1000	0.027	0.027	-2,43
PCSK7	-7,33	0.001996	0.04489	1	1000	0.027	0.027	-1,57
MYH14	-7,37	0.001996	0.04489	1	1000	0.021	0.021	-2,37
LPAR5	-7,46	0.001996	0.04489	1	1000	0.017	0.017	-1,84
FAM46A	-7,48	0.001996	0.04489	1	1000	0.016	0.016	-1,96
VSIG2	-7,52	0.001996	0.04489	1	1000	0.015	0.015	-3,48
LOC729966	-7,54	0.001996	0.04489	1	1000	0.015	0.015	-2,71
CYP4F12	-7,59	0.001996	0.04489	1	1000	0.014	0.014	-2,37
PIP5K1B	-7,68	0.001996	0.04489	1	1000	0.011	0.011	-3,96
RHBDL2	-7,72	0.001996	0.04489	1	1000	0.011	0.011	-2,48
IQGAP2	-7,77	0.001996	0.04489	1	1000	0.0090	0.0090	-4,41
MUC3A	-7,82	0.001996	0.04489	1	1000	0.0090	0.0090	-4,45
SSBP3	-7,89	0.001996	0.04489	1	1000	0.0080	0.0080	-1,64
C1GALT1	-7,96	0.001996	0.04489	1	1000	0.0070	0.0070	-1,64
AGR3	-7,97	0.001996	0.04489	1	1000	0.0070	0.0070	-4,13
ENST00000575279	-7,97	0.001996	0.04489	1	1000	0.0070	0.0070	-1,57
SHROOM3	-8	0.001996	0.04489	1	1000	0.0060	0.0060	-2,48
LYZ	-8,01	0.001996	0.04489	1	1000	0.0060	0.0060	-4,27
TFF1	-8,08	0.001996	0.04489	1	1000	0.0040	0.0040	-6,04
SORT1	-8,17	0.001996	0.04489	1	1000	0.0040	0.0040	-2,07
CYP3A5	-8,19	0.001996	0.04489	1	1000	0.0030	0.0030	-3,42
BC044939	-8,19	0.001996	0.04489	1	1000	0.0030	0.0030	-1,35
SDCBP2	-8,21	0.001996	0.04489	1	1000	0.0030	0.0030	-2,03
TNK1	-8,35	0.001996	0.04489	1	1000	0.0020	0.0020	-1,29
AGR2	-8,4	0.001996	0.04489	1	1000	0.0020	0.0020	-3,19
SCP2	-8,42	0.001996	0.04489	1	1000	0.0020	0.0020	-1,61
CLDN18	-8,52	0.001996	0.04489	1	1000	0.0010	0.0010	-7,24
LIPH	-8,54	0.001996	0.04489	1	1000	0.0010	0.0010	-3,04
TMEM45B	-8,61	0.001996	0.04489	1	1000	0.0010	0.0010	-2,72
MUC13	-8,76	0.001996	0.04489	1	1000	0	0	-5,09
POF1B	-8,77	0.001996	0.04489	1	1000	0	0	-3,69
PLAC8	-8,88	0.001996	0.04489	1	1000	0	0	-3,44
OTTHUMG00000169357	-8,94	0.001996	0.04489	1	1000	0	0	-5,29
C9orf152	-8,96	0.001996	0.04489	1	1000	0	0	-2,33
CAPN5	-9	0.001996	0.04489	1	1000	0	0	-2,79
OTTHUMG00000130102	-9,12	0.001996	0.04489	1	1000	0	0	-4,51
FUT2	-9,17	0.001996	0.04489	1	1000	0	0	-2,94
PRR15	-9,21	0.001996	0.04489	1	1000	0	0	-2,79
AATK	-9,48	0.001996	0.04489	1	1000	0	0	-1,29
n367191	-9,51	0.001996	0.04489	1	1000	0	0	-5,34
PRR15L	-9,69	0.001996	0.04489	1	1000	0	0	-3,64
BCL2L15	-9,71	0.001996	0.04489	1	1000	0	0	-4,75
TSPAN8	-10,2	0.001996	0.04489	1	1000	0	0	-4,62
ERN2	-10,83	0.001996	0.04489	1	1000	0	0	-3,86
CTSE	-13,67	0.001996	0.04489	1	1000	0	0	-5,42

Supplementary Table 3

NAME	SIZE	ES	NES	NOM p-val	FDR q-val	FWER p-val	RANK AT MAX	LEADING EDGE
E2F_01	65	0,71688604	2,069001	0	0,001072016	0,001	5505	tags=63%, list=16%, signal=75%
E2F_Q3	219	0,62383306	2,044898	0	0,001072016	0,001	4851	tags=49%, list=14%, signal=57%
MAX_01	246	0,56084824	2,0318317	0	7,15E-04	0,001	6406	tags=48%, list=19%, signal=58%
SGCGSSAAA_E2F1DP2_01	164	0,6777425	2,0167549	0	5,36E-04	0,001	5363	tags=62%, list=16%, signal=73%
NRF1_Q6	230	0,59901047	2,0151029	0	4,29E-04	0,001	7122	tags=57%, list=21%, signal=72%
E2F1_Q3	232	0,6206008	2,0109532	0	3,57E-04	0,001	6059	tags=56%, list=18%, signal=67%
E2F1_Q6_01	232	0,65396297	2,0094018	0	3,06E-04	0,001	5937	tags=62%, list=17%, signal=74%
E2F4DP1_01	231	0,64012724	2,004981	0	2,68E-04	0,001	5916	tags=58%, list=17%, signal=70%
E2F_03	235	0,618022	2,0043006	0	2,38E-04	0,001	5276	tags=52%, list=15%, signal=61%
E2F1DP1RB_01	223	0,6220678	1,9915024	0	2,14E-04	0,001	5266	tags=54%, list=15%, signal=63%
E2F_02	225	0,644968	1,9887927	0	1,95E-04	0,001	5916	tags=60%, list=17%, signal=72%
E2F_Q4_01	224	0,6426877	1,9885998	0	1,79E-04	0,001	5099	tags=56%, list=15%, signal=65%
E2F1DP1_01	225	0,6426542	1,9852487	0	1,65E-04	0,001	5916	tags=59%, list=17%, signal=71%
E2F1DP2_01	225	0,6426542	1,9852487	0	1,53E-04	0,001	5916	tags=59%, list=17%, signal=71%
E2F4DP2_01	225	0,6426542	1,9852487	0	1,43E-04	0,001	5916	tags=59%, list=17%, signal=71%
E2F_Q4	229	0,64235616	1,9757106	0	1,34E-04	0,001	4851	tags=54%, list=14%, signal=63%
AACYNNNNTTCCS_UNKNOWN	89	0,6020581	1,9742961	0	1,26E-04	0,001	7425	tags=56%, list=22%, signal=72%
E2F_Q6_01	230	0,59685165	1,9722219	0	1,73E-04	0,002	6059	tags=53%, list=18%, signal=65%
E2F1_Q4	231	0,57277083	1,9718378	0	1,64E-04	0,002	5617	tags=47%, list=16%, signal=56%
AHR_Q5	202	0,49665168	1,9697707	0	1,56E-04	0,002	6674	tags=47%, list=20%, signal=58%
E2F1_Q6	225	0,6497277	1,9667757	0	1,48E-04	0,002	6059	tags=61%, list=18%, signal=74%
YGCGYRCGC_UNKNOWN	308	0,50707746	1,9635837	0	1,42E-04	0,002	6500	tags=45%, list=19%, signal=56%
E2F_Q3_01	225	0,61605215	1,9585847	0	1,35E-04	0,002	4891	tags=52%, list=14%, signal=60%
E2F_Q6	227	0,63767177	1,9531072	0	2,11E-04	0,003	5363	tags=56%, list=16%, signal=66%
ARNT_01	243	0,52632076	1,95166	0	2,02E-04	0,003	6406	tags=45%, list=19%, signal=55%
USF_01	242	0,554708	1,942928	0	8,56E-04	0,006	6211	tags=48%, list=18%, signal=58%
E2F1_Q4_01	219	0,61183363	1,9375268	0	9,55E-04	0,008	4891	tags=52%, list=14%, signal=60%
ARNT_02	233	0,5552773	1,9360294	0	0,001027415	0,01	7145	tags=52%, list=21%, signal=65%
E2F1_Q3_01	237	0,48309308	1,933588	0	9,92E-04	0,01	5646	tags=41%, list=17%, signal=49%
RRCCGTTA_UNKNOWN	83	0,61309725	1,9301039	0	9,59E-04	0,01	5288	tags=53%, list=16%, signal=63%
MYCMAX_03	236	0,50281674	1,8915553	0	0,002270813	0,024	6625	tags=44%, list=19%, signal=54%
GCCATNTTG_YY1_Q6	403	0,561281	1,8912135	0	0,00219985	0,024	6674	tags=49%, list=20%, signal=60%
MYCMAX_01	244	0,50696	1,889032	0	0,002133188	0,024	6406	tags=46%, list=19%, signal=56%
GABP_B	245	0,58180326	1,8830209	0	0,002070447	0,024	6076	tags=50%, list=18%, signal=60%
KTGGYRSGAA_UNKNOWN	73	0,62407196	1,8740988	0	0,002155614	0,026	5088	tags=51%, list=15%, signal=59%
USF_C	263	0,47580582	1,873097	0	0,002095736	0,026	7164	tags=47%, list=21%, signal=59%
MYCMAX_02	249	0,46503413	1,8689786	0	0,00217986	0,029	6531	tags=40%, list=19%, signal=49%
KRCTCNNNNMANAGC_UNKNOWN	52	0,66755724	1,8657099	0,004405286	0,002122495	0,029	5732	tags=60%, list=17%, signal=72%
MYCMAX_B	250	0,4625654	1,8654294	0	0,002068072	0,029	8243	tags=46%, list=24%, signal=60%
HIF1_Q5	235	0,4722556	1,8644601	0	0,002044563	0,029	6402	tags=41%, list=19%, signal=50%
YY1_Q6	225	0,5304859	1,8440818	0	0,002696399	0,045	6674	tags=49%, list=20%, signal=61%
USF_Q6	244	0,4831221	1,8438641	0	0,002660392	0,046	7145	tags=48%, list=21%, signal=60%
KMCATNNWGGA_UNKNOWN	85	0,5449627	1,8412355	0	0,002645234	0,047	9273	tags=64%, list=27%, signal=87%
USF_02	260	0,47701502	1,8410499	0	0,002612026	0,047	6543	tags=45%, list=19%, signal=55%
GKCGCNNNNNNNTGAYG_UNKNOWN	56	0,70753175	1,8381547	0	0,002642296	0,049	5842	tags=71%, list=17%, signal=86%
GGGNRMNNYCAT_UNKNOWN	78	0,49553823	1,8367819	0	0,002584855	0,049	4624	tags=35%, list=14%, signal=40%
ELK1_02	240	0,5757288	1,8354293	0	0,002555109	0,049	6063	tags=49%, list=18%, signal=59%
HIF1_Q3	218	0,48657522	1,8314209	0	0,003032013	0,054	6353	tags=44%, list=19%, signal=53%
TCCCRNNRTGC_UNKNOWN	202	0,5406609	1,814745	0	0,003503693	0,063	5422	tags=46%, list=16%, signal=54%
NMYC_01	260	0,47277105	1,8143542	0	0,003433619	0,063	7164	tags=47%, list=21%, signal=59%
ACTAYRNNNCCCR_UNKNOWN	427	0,52404714	1,8096784	0	0,003735126	0,068	6845	tags=48%, list=20%, signal=59%
CCAWNWWNNNGGC_UNKNOWN	79	0,49038947	1,8066221	0	0,00374049	0,069	6516	tags=46%, list=19%, signal=56%
USF2_Q6	239	0,49415028	1,8041229	0,004750594	0,003803395	0,071	6312	tags=47%, list=19%, signal=58%
YYCATTCAWW_UNKNOWN	186	0,4553238	1,7974367	0	0,004009638	0,078	8204	tags=46%, list=24%, signal=60%
NFMUE1_Q6	227	0,5242831	1,7871	0	0,004542024	0,083	5722	tags=44%, list=17%, signal=52%
CCAATNNSNNNGCG_UNKNOWN	55	0,53639096	1,7844456	0	0,004624021	0,084	7425	tags=56%, list=22%, signal=72%
YY1_02	233	0,5015169	1,7808806	0	0,004748763	0,087	6674	tags=45%, list=20%, signal=56%
GGAANCGGAANY_UNKNOWN	100	0,5758505	1,780706	0	0,004694612	0,087	4983	tags=44%, list=15%, signal=51%
PPARG_01	42	0,60740036	1,7755065	0,002347418	0,004762779	0,089	3124	tags=43%, list=9%, signal=47%
TMTCGCGANR_UNKNOWN	151	0,59017414	1,7688471	0,006637168	0,00526506	0,095	7385	tags=56%, list=22%, signal=71%
NRF2_01	252	0,5365831	1,7680593	0	0,005200648	0,095	6063	tags=47%, list=18%, signal=57%
CETS1P54_01	239	0,51208746	1,7675267	0	0,005173057	0,096	8484	tags=57%, list=25%, signal=75%
MYC_Q2	176	0,48993844	1,7671808	0,004819277	0,005090945	0,096	6312	tags=47%, list=19%, signal=58%
ACTWSNACTNY_UNKNOWN	97	0,47076142	1,7644967	0,002309469	0,005144922	0,097	7618	tags=43%, list=22%, signal=56%
PAX4_01	247	0,40713295	1,7642201	0	0,005065769	0,097	5880	tags=34%, list=17%, signal=41%
SP1_Q4_01	243	0,42883888	1,7598888	0	0,005503181	0,1	7804	tags=46%, list=23%, signal=59%
AAGWWRNYGGC_UNKNOWN	111	0,5319752	1,7597982	0	0,005421044	0,1	5505	tags=46%, list=16%, signal=55%
SP1_Q6	242	0,43377686	1,7592078	0	0,005385077	0,101	8046	tags=48%, list=24%, signal=63%
GATA1_01	237	0,38630062	1,7580023	0	0,005374055	0,101	6296	tags=30%, list=18%, signal=37%
GGCNRNWCTTYS_UNKNOWN	77	0,5284394	1,7514237	0,007058824	0,006145108	0,113	6078	tags=44%, list=18%, signal=54%
WCTCNATGGY_UNKNOWN	81	0,50804037	1,7496974	0,002217295	0,006238401	0,118	7959	tags=51%, list=23%, signal=66%
AP2_Q6	250	0,41489512	1,7484306	0	0,006185187	0,118	6240	tags=37%, list=18%, signal=45%
USF_Q6_01	219	0,44729006	1,7465669	0	0,00624602	0,12	6812	tags=42%, list=20%, signal=53%
CGGAARNGGCNG_UNKNOWN	47	0,5788362	1,7424079	0,006369427	0,006533946	0,127	5034	tags=43%, list=15%, signal=50%
ZF5_B	231	0,4071374	1,741617	0	0,006505332	0,127	6402	tags=35%, list=19%, signal=42%
CREB_Q3	237	0,45116696	1,738479	0	0,006555098	0,129	5659	tags=38%, list=17%, signal=45%
ZF5_01	226	0,43346012	1,736104	0,007352941	0,006785811	0,13	6320	tags=41%, list=19%, signal=50%
MCAATNNNNNGCG_UNKNOWN	81	0,4593358	1,734409	0,002386635	0,00679207	0,13	5722	tags=37%, list=17%, signal=44%
GATGKMRGCG_UNKNOWN	64	0,5625269	1,7325737	0,002173913	0,007007267	0,131	5479	tags=52%, list=16%, signal=61%
SP1_Q6_01	234	0,42785543	1,7258936	0,002386635	0,007403362	0,14	5216	tags=35%, list=15%, signal=41%
SREBP1_01	161	0,5268156	1,7255791	0,004716981	0,007343082	0,141	8358	tags=58%, list=25%, signal=76%
CGTSACG_PAX3_B	142	0,45867077	1,723281	0,00243309	0,007622446	0,143	5920	tags=39%, list=17%, signal=47%
GCGNNANTTCC_UNKNOWN	112	0,5022811	1,7062771	0,006818182	0,009139366	0,156	7013	tags=51%, list=21%, signal=64%
AACWWCAANK_UNKNOWN	135	0,45860323	1,7018266	0	0,009405605	0,162	7181	tags=44%, list=21%, signal=55%
CEBPGAMMA_Q6	242	0,37448654	1,7009857	0	0,009384413	0,164	5594	tags=31%, list=16%, signal=37%
NFY_Q6	248	0,3895214	1,6938889	0	0,010136286	0,169	5525	tags=33%, list=16%, signal=40%
MTF1_Q4	237	0,3803764	1,6905315	0	0,010496361	0,172	5916	tags=34%, list=17%, signal=41%
TAANNYSGCG_UNKNOWN	75	0,5530562	1,6884001	0,008888889	0,010458443	0,173	5458	tags=45%, list=16%, signal=54%
SP1_Q2_01	234	0,40182292	1,686874	0,002631579	0,010476857	0,174	8509	tags=45%, list=25%, signal=59%
TTCYRGAA_UNKNOWN	312	0,3948789	1,6857613	0	0,010587732	0,177	7275	tags=38%, list=21%, signal=48%
CMYB_01	230	0,4314796	1,6834916	0,002450981	0,010861618	0,185	6878	tags=42%, list=20%, signal=52%
YRTCANNRCGC_UNKNOWN	65	0,5147306	1,681201	0,004950495	0,011089921	0,188	6802	tags=46%, list=20%, signal=58%
CREB_01	251	0,42575783	1,6787407	0	0,011193953	0,192	6223	tags=36%, list=18%, signal=44%
TTCNRGNNNNTTC_HSF_Q6	142	0,44004273	1,6747116	0,006896552	0,011433153	0,195	4970	tags=32%, list=15%, signal=38%
GTCNYYATGR_UNKNOWN	104	0,43900207	1,6736637	0	0,011482854	0,196	6759	tags=41%, list=20%, signal=51%
NFY_Q6_01	247	0,41741443	1,6699846	0,004987531	0,011915324	0,206	6211	tags=35%, list=18%, signal=43%
ERR1_Q2	251	0,38004556	1,6691236	0	0,011897522	0,208	5306	tags=30%, list=16%, signal=35%
GGCNKCCATNK_UNKNOWN	110	0,49471205	1,6681781	0,002237137	0,011854652	0,209	6920	tags=44%, list=20%, signal=55%
STAT1_03	237	0,39914688	1,6680492	0	0,01176268	0,209	7495	tags=39%, list=22%, signal=50%
TGACGTCA_ATF3_Q6	221	0,4165846	1,6676539	0,002544529	0,011689782	0,209	6223	tags=35%, list=18%, signal=42%
NFY_C	232	0,3900325	1,6628846	0	0,012155616	0,218	6147	tags=34%, list=18%, signal=41%
CREBP1CJUN_01	248	0,4108944	1,6565487	0	0,012806525	0,225	6223	tags=35%, list=18%, signal=43%
SF1_Q6	247	0,39826733	1,654207	0,004926108	0,013028029	0,23	4709	tags=31%, list=14%, signal=35%
CHOP_01	227	0,38729033	1,6539664	0	0,012915944	0,23	7731	tags=37%, list=23%, signal=48%
ACAWNRNSRCGG_UNKNOWN	59	0,50257057	1,6529454	0,006696429	0,01290275	0,231	8290	tags=53%, list=24%, signal=69%
ATCMNTCCGY_UNKNOWN	48	0,54046476	1,6513451	0,011185682	0,012943705	0,232	9253	tags=58%, list=27%, signal=80%
GCGSCMNTTT_UNKNOWN	68	0,5473663	1,649217	0,010893246	0,013097855	0,234	4970	tags=44%, list=15%, signal=52%
ATGGYGGA_UNKNOWN	95	0,47432935	1,64204	0,010989011	0,014170366	0,244	5418	tags=38%, list=16%, signal=45%
KCCGNSWTTT_UNKNOWN	96	0,47615972	1,636701	0,009280742	0,014907151	0,254	8290	tags=55%, list=24%, signal=73%
E4F1_Q6	276	0,39943805	1,6346606	0	0,015026361	0,256	7602	tags=42%, list=22%, signal=54%
STAT1_02	240	0,41169634	1,634338	0,002638523	0,014987301	0,257	7295	tags=40%, list=21%, signal=50%
EGR1_01	259	0,37538776	1,6314634	0	0,015184326	0,26	6625	tags=35%, list=19%, signal=43%
TGACCTTG_SF1_Q6	237	0,38964114	1,6310734	0	0,015133272	0,261	5176	tags=30%, list=15%, signal=36%
GGCNNMSMYNTTG_UNKNOWN	71	0,4481026	1,6260009	0,007058824	0,016379284	0,276	5674	tags=42%, list=17%, signal=51%
CCGNMNNTNACG_UNKNOWN	73	0,5394079	1,6216178	0,034246575	0,017026411	0,283	7843	tags=55%, list=23%, signal=71%
MSX1_01	169	0,36575627	1,6181542	0,003472222	0,017404433	0,286	8626	tags=39%, list=25%, signal=52%
CREB_Q4_01	202	0,41752693	1,6126719	0,007481297	0,018454801	0,299	6349	tags=36%, list=19%, signal=44%
CEBP_Q2	224	0,3620475	1,6113887	0	0,01860302	0,304	6061	tags=30%, list=18%, signal=37%
CREB_02	239	0,4268587	1,6102782	0,007425743	0,018645536	0,305	6224	tags=39%, list=18%, signal=47%
GGCKCATGS_UNKNOWN	63	0,4295359	1,6096823	0,007281554	0,018557986	0,306	5511	tags=33%, list=16%, signal=40%
ELK1_01	257	0,39687753	1,6094828	0	0,018423794	0,306	7124	tags=40%, list=21%, signal=50%
NRF2_Q4	244	0,36847663	1,6091505	0,005988024	0,018331304	0,306	6678	tags=33%, list=20%, signal=41%
EVI1_06	21	0,5520367	1,6080633	0,025510205	0,018358065	0,307	7997	tags=43%, list=23%, signal=56%
CREB_Q2_01	210	0,40767515	1,6054218	0,002427185	0,018606644	0,313	6211	tags=35%, list=18%, signal=42%
EGR_Q6	262	0,355086	1,6004477	0,002680965	0,019607248	0,324	7013	tags=34%, list=21%, signal=42%
GATA2_01	102	0,39059368	1,6000158	0,00243309	0,019474505	0,324	6825	tags=34%, list=20%, signal=43%
PAX3_01	20	0,5245109	1,5942917	0,030800821	0,020254353	0,333	5722	tags=40%, list=17%, signal=48%

YTCCCRNNAGGY_UNKNOWN	66	0,4475802	1,5878838	0,005194805	0,021422375	0,344	7022	tags=42%, list=21%, signal=53%
TEL2_Q6	224	0,41210398	1,5842541	0,002314815	0,02193649	0,349	8134	tags=46%, list=24%, signal=59%
NGFIC_01	243	0,36928576	1,5832875	0	0,021990536	0,351	6486	tags=33%, list=19%, signal=41%
HSF_Q6	186	0,36061206	1,5797915	0,01058201	0,022457061	0,357	7442	tags=38%, list=22%, signal=48%
TTTNNANAGCYR_UNKNOWN	120	0,40997502	1,574354	0,016666668	0,023336124	0,366	5992	tags=35%, list=18%, signal=42%
AP2_Q3	246	0,35111576	1,5672379	0	0,024587832	0,377	6378	tags=31%, list=19%, signal=38%
EGR3_01	82	0,40061373	1,5668904	0,002710027	0,024585804	0,381	6443	tags=37%, list=19%, signal=45%
RREB1_01	200	0,35006633	1,5644774	0	0,024992807	0,384	6809	tags=32%, list=20%, signal=40%
MYAATNNNNNNNGGC_UNKNOWN	106	0,37262964	1,5616213	0,013227513	0,025382021	0,388	6011	tags=32%, list=18%, signal=39%
TCANNTGAY_SREBP1_01	451	0,37771165	1,5589868	0,005235602	0,025811547	0,39	7145	tags=37%, list=21%, signal=46%
CREB_Q4	254	0,37081173	1,5531402	0,002544529	0,027096251	0,399	6349	tags=34%, list=19%, signal=41%
YWATTWNNRGCT_UNKNOWN	64	0,39964128	1,5502336	0,006006006	0,027728008	0,408	4888	tags=30%, list=14%, signal=35%
SMTTTTGT_UNKNOWN	390	0,34465975	1,5494343	0	0,027664546	0,408	7868	tags=36%, list=23%, signal=47%
SYATTGTG_UNKNOWN	221	0,34168547	1,5419697	0,002923977	0,028949717	0,415	8188	tags=38%, list=24%, signal=49%
STAT_01	241	0,34695527	1,5411272	0,003205128	0,028865108	0,415	8412	tags=38%, list=25%, signal=50%
WHN_B	240	0,35477656	1,5404385	0,002881844	0,028829884	0,416	7512	tags=36%, list=22%, signal=46%
YGCANTGCR_UNKNOWN	119	0,3871926	1,5359821	0,02710027	0,029781519	0,426	8693	tags=43%, list=25%, signal=57%
RYAAAKNNNNNNTTGW_UNKNOWN	82	0,37797058	1,5356637	0,011267605	0,029693715	0,426	5160	tags=30%, list=15%, signal=36%
TGASTMAGC_NFE2_01	185	0,37540647	1,5356407	0,019444445	0,029498372	0,427	5841	tags=31%, list=17%, signal=38%
SNACANNNYSYAGA_UNKNOWN	84	0,43850568	1,5345253	0,016509434	0,029517721	0,429	8416	tags=45%, list=25%, signal=60%
PR_02	133	0,36456034	1,5342051	0	0,029409023	0,429	5762	tags=33%, list=17%, signal=40%
PAX6_01	94	0,3544178	1,5312206	0,00660066	0,029782623	0,434	4328	tags=22%, list=13%, signal=26%
AP1_Q4	262	0,36125696	1,5286549	0,029498525	0,030168673	0,44	6950	tags=34%, list=20%, signal=42%
AP2_Q6_01	259	0,3529117	1,5280099	0,022959184	0,030103216	0,441	5418	tags=29%, list=16%, signal=34%
NFY_01	240	0,35857385	1,5262463	0,002666667	0,030237574	0,441	6328	tags=32%, list=19%, signal=39%
ATF_B	179	0,36286867	1,52459	0,008571428	0,030559769	0,445	6223	tags=32%, list=18%, signal=39%
TITF1_Q3	225	0,33356524	1,5214187	0,003311258	0,031242983	0,45	5842	tags=30%, list=17%, signal=36%
YAATNANRNNNCAG_UNKNOWN	63	0,3698694	1,5194733	0,020771513	0,031577826	0,451	7608	tags=41%, list=22%, signal=53%
TNCATNTCCYR_UNKNOWN	126	0,40578648	1,516853	0,013089005	0,031989038	0,455	8769	tags=44%, list=26%, signal=60%
STAT3_02	136	0,35184914	1,51021	0,003030303	0,033643305	0,468	8359	tags=43%, list=25%, signal=56%
LXR_Q3	73	0,3842369	1,5076554	0,016348774	0,034208935	0,472	8427	tags=44%, list=25%, signal=58%
NFE2_01	260	0,34470862	1,5013247	0,017857144	0,035655543	0,481	8042	tags=37%, list=24%, signal=47%
GGAMTNNNNNTCCY_UNKNOWN	109	0,41017327	1,4990107	0,038647342	0,036063407	0,483	6076	tags=37%, list=18%, signal=45%
YTTCCNNNGGAMR_UNKNOWN	52	0,39282715	1,4982064	0,015706806	0,03606502	0,484	7079	tags=31%, list=21%, signal=39%
STAT1_01	64	0,42128453	1,4981588	0,027295286	0,03585426	0,484	7272	tags=45%, list=21%, signal=57%
CCCNNNNNNAAGWT_UNKNOWN	93	0,40472123	1,4958724	0,02538071	0,03633341	0,489	6672	tags=43%, list=20%, signal=53%
YY1_01	236	0,3461161	1,488	0,010928961	0,038850527	0,503	7045	tags=35%, list=21%, signal=44%
MYB_Q3	229	0,33651048	1,4844161	0,008498584	0,039728384	0,509	8609	tags=41%, list=25%, signal=55%
FOXO3_01	228	0,32590142	1,4805843	0,003448276	0,040921614	0,512	8133	tags=36%, list=24%, signal=47%
CREB_Q2	250	0,34439707	1,4792608	0,010810811	0,041196812	0,515	5940	tags=30%, list=17%, signal=37%
SP3_Q3	234	0,33605063	1,4763021	0,002898551	0,04213895	0,522	6759	tags=34%, list=20%, signal=42%
CATTGTYY_SOX9_B1	348	0,32424015	1,4757936	0,015527951	0,042049944	0,523	6077	tags=31%, list=18%, signal=38%
AHR_01	56	0,40014595	1,4739085	0,033254158	0,042609286	0,531	6714	tags=43%, list=20%, signal=53%
TGANNYRGCA_TCF11MAFG_01	292	0,31889263	1,4726244	0,003322259	0,04282875	0,533	6646	tags=32%, list=19%, signal=39%
EGR2_01	190	0,3201042	1,4690992	0,006430868	0,043962862	0,539	5675	tags=27%, list=17%, signal=33%
PAX2_01	53	0,35889533	1,4685419	0,014124294	0,04387384	0,539	3914	tags=26%, list=11%, signal=30%
GCTNWTTGK_UNKNOWN	289	0,32628274	1,4672571	0,003144654	0,04409459	0,543	6029	tags=28%, list=18%, signal=34%
WWTAAGGC_UNKNOWN	133	0,32924506	1,4626136	0,006514658	0,04534333	0,546	7319	tags=36%, list=21%, signal=46%
MYB_Q5_01	246	0,335059	1,4613937	0,015576324	0,045492973	0,548	9663	tags=46%, list=28%, signal=64%
AHRARNT_01	134	0,3458286	1,459769	0,00955414	0,045686733	0,55	8427	tags=40%, list=25%, signal=52%
ALPHACP1_01	246	0,33482495	1,4593894	0,005830904	0,04564333	0,551	6248	tags=30%, list=18%, signal=37%
TAXCREB_01	129	0,33783293	1,4563662	0,00867052	0,046444003	0,559	6224	tags=32%, list=18%, signal=39%
AP2ALPHA_01	224	0,3442359	1,4501178	0,03324808	0,048866812	0,568	9401	tags=47%, list=28%, signal=64%
CREBP1_Q2	236	0,33408007	1,4471873	0,014662757	0,049662277	0,572	6349	tags=31%, list=19%, signal=38%
SP1_01	229	0,3278256	1,442458	0,018867925	0,05111656	0,576	7329	tags=36%, list=21%, signal=46%
AR_03	58	0,40057704	1,4409857	0,04589372	0,051370904	0,58	8059	tags=45%, list=24%, signal=59%
ATF_01	244	0,32877272	1,440415	0,014577259	0,051421363	0,581	7705	tags=34%, list=23%, signal=44%
BACH2_01	259	0,337829	1,4371092	0,041543026	0,05253631	0,587	7804	tags=39%, list=23%, signal=50%
GR_Q6_01	262	0,313974	1,4356501	0,009287925	0,05298594	0,592	8036	tags=38%, list=24%, signal=49%
ER_Q6_02	242	0,3120098	1,4335885	0,017857144	0,053533327	0,598	6972	tags=33%, list=20%, signal=41%
AR_02	40	0,40587962	1,4308176	0,031496063	0,05427517	0,6	5478	tags=35%, list=16%, signal=42%
PAX4_04	208	0,31598544	1,428647	0,012738854	0,055101585	0,602	5722	tags=27%, list=17%, signal=33%
WTTGKCTG_UNKNOWN	482	0,2965241	1,4244989	0,009463723	0,056669205	0,608	6996	tags=32%, list=21%, signal=39%
MEIS1_01	233	0,314183	1,420789	0,010380623	0,057791863	0,609	6746	tags=31%, list=20%, signal=38%
AR_Q6	247	0,29327273	1,4180367	0,012903226	0,05887052	0,616	5629	tags=26%, list=17%, signal=30%
CDX2_Q5	235	0,2954074	1,4179814	0,011764706	0,058578365	0,616	5992	tags=26%, list=18%, signal=32%
SMAD_Q6	238	0,3072031	1,417484	0,019169329	0,05840983	0,616	6650	tags=29%, list=19%, signal=36%
CEBP_C	195	0,3244453	1,4132599	0,009118541	0,06000708	0,625	6537	tags=31%, list=19%, signal=38%
STAT6_01	255	0,3135843	1,4130193	0,010204081	0,059747707	0,625	8046	tags=33%, list=24%, signal=43%
RGAANNTTC_HSF1_01	420	0,31401417	1,4116561	0,030674847	0,060275767	0,63	8192	tags=39%, list=24%, signal=51%
HMX1_01	39	0,4066662	1,4087865	0,11464968	0,061307896	0,638	1687	tags=18%, list=5%, signal=19%
AP1FJ_Q2	256	0,32123175	1,4066852	0,049844235	0,062143385	0,646	6950	tags=31%, list=20%, signal=39%
SOX9_B1	229	0,31970638	1,4027687	0,022151899	0,06390347	0,657	5884	tags=28%, list=17%, signal=34%
TGIF_01	236	0,31899202	1,402059	0,009615385	0,06390059	0,657	9633	tags=47%, list=28%, signal=65%
PPAR_DR1_Q2	252	0,33205903	1,4019347	0,02247191	0,06365813	0,657	6191	tags=29%, list=18%, signal=36%
AP1_Q2	255	0,32260764	1,4007518	0,051829267	0,06400331	0,66	7804	tags=34%, list=23%, signal=43%
HSF1_01	251	0,30386552	1,3992538	0,013937282	0,06451137	0,662	8217	tags=39%, list=24%, signal=51%
GCM_Q2	231	0,29396445	1,3975105	0,020761246	0,06488137	0,664	7825	tags=35%, list=23%, signal=45%
GTTNYYNNGGTNA_UNKNOWN	86	0,38712886	1,3956656	0,08158508	0,06557048	0,669	5187	tags=33%, list=15%, signal=38%
RORA1_01	231	0,29852247	1,3897817	0,01369863	0,06800372	0,675	6646	tags=29%, list=19%, signal=36%
MYOD_01	252	0,2971173	1,381705	0,016286645	0,07240309	0,691	6273	tags=29%, list=18%, signal=35%
HMGIY_Q6	238	0,30444348	1,3815191	0,013793103	0,07223061	0,691	8694	tags=38%, list=25%, signal=50%
NFAT_Q6	237	0,30425555	1,3797709	0,022653721	0,07308557	0,697	5239	tags=25%, list=15%, signal=30%
CEBPB_02	248	0,31707156	1,3792115	0,016286645	0,07295574	0,697	6283	tags=30%, list=18%, signal=36%
FAC1_01	214	0,3086647	1,3776623	0,030487806	0,07355178	0,702	6355	tags=29%, list=19%, signal=35%
CEBPB_01	251	0,2989556	1,3764286	0,020689655	0,07377748	0,705	6646	tags=29%, list=19%, signal=36%
YNGTTNNNATT_UNKNOWN	350	0,28808483	1,3704995	0,007462686	0,076733716	0,714	5583	tags=24%, list=16%, signal=29%
ATF1_Q6	220	0,29713213	1,3691504	0,015384615	0,077268116	0,717	7602	tags=31%, list=22%, signal=40%
TCF11MAFG_01	196	0,31056976	1,3687679	0,05	0,0770324	0,717	6312	tags=29%, list=19%, signal=35%
AR_01	150	0,2950479	1,364242	0,0147929	0,07955756	0,722	6932	tags=33%, list=20%, signal=41%
MYOD_Q6_01	247	0,2897648	1,3632936	0,029069768	0,07974326	0,723	4947	tags=23%, list=15%, signal=27%
OCT1_07	155	0,3024976	1,361489	0,032894738	0,08051942	0,726	5866	tags=25%, list=17%, signal=29%
EFC_Q6	254	0,28694803	1,3610686	0,026548672	0,08029707	0,726	8087	tags=35%, list=24%, signal=45%
IRF2_01	116	0,33031505	1,3604733	0,042071197	0,08053623	0,728	4316	tags=22%, list=13%, signal=25%
COUP_DR1_Q6	234	0,32024196	1,3585255	0,039893616	0,081264496	0,731	5418	tags=25%, list=16%, signal=30%
SOX5_01	251	0,30659372	1,3581344	0,041666668	0,08107966	0,731	6169	tags=27%, list=18%, signal=33%
NERF_Q2	233	0,298259	1,3568796	0,015625	0,0816442	0,731	8207	tags=38%, list=24%, signal=49%
COUP_01	250	0,30547065	1,3548417	0,017492712	0,08258864	0,737	5418	tags=26%, list=16%, signal=30%
TFIIA_Q6	241	0,2869172	1,3506738	0,019736841	0,08541374	0,748	6248	tags=28%, list=18%, signal=34%
NKX22_01	179	0,29330572	1,3492519	0,018181818	0,085775696	0,749	5369	tags=26%, list=16%, signal=31%
LXR_DR4_Q3	89	0,32442254	1,348623	0,06596306	0,08586449	0,751	3382	tags=18%, list=10%, signal=20%
AP4_01	249	0,27905655	1,3480636	0,03345725	0,08587941	0,751	7573	tags=34%, list=22%, signal=43%
DR1_Q3	245	0,31400636	1,3470654	0,047222223	0,085984364	0,752	6191	tags=28%, list=18%, signal=34%
AAAYRNCTG_UNKNOWN	355	0,28638342	1,3467005	0,01509434	0,085777216	0,752	5187	tags=23%, list=15%, signal=27%
MIF1_01	174	0,33307806	1,3464653	0,08549223	0,08556145	0,753	9613	tags=44%, list=28%, signal=61%
SREBP_Q3	246	0,27651083	1,3460592	0,019607844	0,08531595	0,753	5616	tags=26%, list=16%, signal=31%
CRGAARNNNNCGA_UNKNOWN	47	0,4400654	1,3453654	0,10660981	0,085251346	0,753	3124	tags=30%, list=9%, signal=33%
TST1_01	244	0,28462434	1,3436333	0,037288137	0,086337425	0,757	7984	tags=31%, list=23%, signal=40%
GATA4_Q3	232	0,2850433	1,3417624	0,039215688	0,08702223	0,759	6038	tags=27%, list=18%, signal=32%
WCAANNNYCAG_UNKNOWN	238	0,31023327	1,3413653	0,038356163	0,086811304	0,76	6932	tags=34%, list=20%, signal=42%
TCF1P_Q6	253	0,31019136	1,3393806	0,04519774	0,087785766	0,763	5157	tags=24%, list=15%, signal=28%
GR_01	196	0,29737818	1,3393186	0,031034483	0,08745142	0,763	8059	tags=37%, list=24%, signal=48%
BACH1_01	249	0,31318116	1,3383981	0,060509555	0,08769902	0,763	5841	tags=30%, list=17%, signal=36%
PAX4_03	242	0,29514202	1,3369939	0,053977273	0,088184565	0,764	5693	tags=27%, list=17%, signal=32%
AREB6_04	242	0,2922613	1,3358779	0,020746889	0,088525444	0,764	5197	tags=26%, list=15%, signal=30%
TTAYRTAA_E4BP4_01	246	0,29679933	1,3353473	0,017006803	0,08848825	0,765	5405	tags=26%, list=16%, signal=31%
CCAWYNNGAAR_UNKNOWN	136	0,3296522	1,3349634	0,07272727	0,088504694	0,766	8574	tags=40%, list=25%, signal=53%
RACTNNRTTTNC_UNKNOWN	120	0,34234774	1,3301158	0,062015504	0,090999246	0,773	8813	tags=42%, list=26%, signal=56%
OSF2_Q6	249	0,28764734	1,3299618	0,050632913	0,09070116	0,774	7389	tags=34%, list=22%, signal=43%
YAATNRNNNYNATT_UNKNOWN	99	0,30855775	1,3288403	0,06428572	0,09116829	0,776	3412	tags=20%, list=10%, signal=22%
ATF4_Q2	245	0,3112814	1,3280909	0,075980395	0,091202505	0,777	5659	tags=26%, list=17%, signal=31%
GCCNNNWTAAR_UNKNOWN	142	0,31147245	1,3250451	0,043771043	0,09256196	0,779	5336	tags=26%, list=16%, signal=31%
STAT5A_04	202	0,28447387	1,3240083	0,017667845	0,09276234	0,779	6925	tags=29%, list=20%, signal=36%
CACCCBINDINGFACTOR_Q6	260	0,2920712	1,3203726	0,034920637	0,094845586	0,785	5813	tags=27%, list=17%, signal=32%
T3R_Q6	239	0,27503425	1,3154881	0,01459854	0,09765374	0,786	7425	tags=28%, list=22%, signal=36%
ZID_01	247	0,27534294	1,3146312	0,029411765	0,097863995	0,787	8479	tags=36%, list=25%, signal=47%
BRN2_01	225	0,27771527	1,312892	0,02749141	0,09880365	0,791	7745	tags=31%, list=23%, signal=40%
CREBP1_01	168	0,31286344	1,3101033	0,066066064	0,10032452	0,794	7157	tags=33%, list=21%, signal=42%
SMAD4_Q6	231	0,28693032	1,3096296	0,053672317	0,10021875	0,794	6983	tags=32%, list=20%, signal=40%
GTTGNYNNRGNAAC_UNKNOWN	101	0,319106	1,3088685	0,07832898	0,1002809	0,795	5284	tags=25%, list=15%, signal=29%
NFAT_Q4_01	257	0,28612408	1,3068966	0,01968504	0,10097854	0,796	6500	tags=30%, list=19%, signal=36%
DR3_Q4	132	0,3014866	1,3013922	0,048929665	0,104681835	0,804	8427	tags=38%, list=25%, signal=50%

PAX_Q6	246	0,27522442	1,2995431	0,039007094	0,105799355	0,806	7743	tags=30%, list=23%, signal=39%
FXR_Q3	109	0,28386372	1,2977371	0,05	0,10683722	0,809	4627	tags=20%, list=14%, signal=23%
YGTCCTTGR_UNKNOWN	94	0,306973	1,2960678	0,08454811	0,107408404	0,81	6109	tags=27%, list=18%, signal=32%
FOXO4_02	247	0,28298527	1,2951528	0,038732395	0,1077208	0,812	8122	tags=35%, list=24%, signal=46%
AP1_Q6_01	255	0,28973106	1,294404	0,06666667	0,10775486	0,812	9747	tags=43%, list=29%, signal=59%
TTCYNRGAA_STAT5B_01	321	0,26559347	1,2941694	0,019305019	0,10748467	0,813	8412	tags=32%, list=25%, signal=42%
COMP1_01	110	0,29638857	1,292236	0,073469386	0,10847777	0,814	7854	tags=37%, list=23%, signal=48%
ER_Q6_01	254	0,27700433	1,2921242	0,04682274	0,10814057	0,814	6223	tags=26%, list=18%, signal=32%
AP2GAMMA_01	236	0,29109126	1,2891368	0,063400574	0,109863624	0,82	6102	tags=30%, list=18%, signal=36%
LYF1_01	256	0,2726995	1,2887204	0,0529595	0,10975976	0,82	7193	tags=29%, list=21%, signal=36%
MAZ_Q6	187	0,32910824	1,287322	0,15347722	0,110452905	0,821	4434	tags=25%, list=13%, signal=28%
CACBINDINGPROTEIN_Q6	231	0,27503088	1,2872957	0,06647399	0,11005814	0,821	6726	tags=28%, list=20%, signal=34%
RRAGTTGT_UNKNOWN	243	0,27530032	1,2849634	0,02112676	0,11118767	0,822	4434	tags=21%, list=13%, signal=24%
CP2_01	249	0,27994636	1,284623	0,078313254	0,11097737	0,822	5041	tags=23%, list=15%, signal=27%
FOXO1_01	230	0,26650062	1,2845308	0,02283105	0,11062297	0,822	6007	tags=23%, list=18%, signal=27%
STAT5A_01	239	0,2618693	1,2834653	0,023904383	0,110940464	0,824	7286	tags=28%, list=21%, signal=35%
NFKB_Q6_01	221	0,29410198	1,2818502	0,08490566	0,111797445	0,826	7684	tags=33%, list=23%, signal=43%
ER_Q6	261	0,26917508	1,2810109	0,059748426	0,11196329	0,827	6223	tags=27%, list=18%, signal=33%
RFX1_01	246	0,3102468	1,2808437	0,11111111	0,11166412	0,827	4711	tags=25%, list=14%, signal=29%
RFX1_02	263	0,29277804	1,280214	0,08682635	0,11182342	0,828	5197	tags=24%, list=15%, signal=28%
IK1_01	264	0,27949488	1,2800108	0,03529412	0,11161047	0,829	8593	tags=35%, list=25%, signal=46%
POU3F2_02	248	0,2735908	1,2798166	0,05703422	0,111312374	0,829	6878	tags=29%, list=20%, signal=36%
MYB_Q6	239	0,2709446	1,2789632	0,06338028	0,11159976	0,831	8059	tags=33%, list=24%, signal=43%
PR_01	142	0,28983843	1,2782307	0,053977273	0,111720055	0,831	8059	tags=33%, list=24%, signal=43%
E4BP4_01	211	0,29185364	1,2778964	0,05792683	0,11164158	0,832	5405	tags=26%, list=16%, signal=30%
STAT5A_02	136	0,28760114	1,2775258	0,04040404	0,11153356	0,833	5535	tags=26%, list=16%, signal=31%
GR_Q6	260	0,26494104	1,2774401	0,03557312	0,11119072	0,833	6468	tags=25%, list=19%, signal=31%
SPZ1_01	217	0,2812729	1,2752603	0,057471264	0,112577505	0,835	8059	tags=35%, list=24%, signal=46%
TCF11_01	243	0,27930123	1,2742238	0,02877698	0,11301484	0,837	6497	tags=27%, list=19%, signal=33%
OCT1_06	250	0,2738672	1,2741098	0,045296166	0,11268614	0,837	6119	tags=26%, list=18%, signal=31%
FOXJ2_01	171	0,26375747	1,2733018	0,03968254	0,113004744	0,839	7201	tags=27%, list=21%, signal=34%
AP1_Q6	250	0,28943717	1,2688442	0,108359136	0,11580798	0,843	7734	tags=32%, list=23%, signal=42%
FOXO4_01	226	0,27286536	1,267165	0,043650795	0,116866864	0,848	6406	tags=26%, list=19%, signal=31%
TEF_Q6	239	0,26507503	1,2664268	0,04819277	0,11721874	0,848	6435	tags=27%, list=19%, signal=33%
TCCATTKW_UNKNOWN	227	0,30809	1,2648879	0,07580175	0,1182115	0,851	5866	tags=27%, list=17%, signal=32%
AMEF2_Q6	243	0,27466547	1,2626284	0,07210031	0,11948731	0,853	7444	tags=32%, list=22%, signal=40%
CAGNYGKNAAA_UNKNOWN	72	0,31245357	1,2621574	0,097765364	0,11939164	0,853	5874	tags=28%, list=17%, signal=33%
STAT_Q6	250	0,25263527	1,2616234	0,019455252	0,11957642	0,855	6932	tags=26%, list=20%, signal=32%
ETS_Q4	237	0,28556436	1,2597427	0,060344826	0,120643765	0,855	6908	tags=30%, list=20%, signal=37%
PPARA_02	122	0,29077697	1,2567657	0,12589073	0,12274629	0,857	5675	tags=26%, list=17%, signal=31%
CEBPA_01	231	0,28765398	1,2549949	0,09859155	0,12396968	0,859	7019	tags=30%, list=21%, signal=37%
NCX_01	160	0,26269254	1,2544461	0,074074075	0,12397486	0,859	7770	tags=29%, list=23%, signal=38%
MZF1_02	222	0,28382984	1,2541229	0,07514451	0,12390401	0,859	5892	tags=26%, list=17%, signal=31%
NFKB_Q6	246	0,28025368	1,2533474	0,11598746	0,124103956	0,86	5994	tags=27%, list=18%, signal=33%
STAT3_01	21	0,40874398	1,2527288	0,16624685	0,12409674	0,861	3228	tags=24%, list=9%, signal=26%
P300_01	239	0,2697366	1,2496282	0,06006006	0,12636349	0,866	6349	tags=26%, list=19%, signal=32%
AP1_Q4_01	251	0,28913835	1,2476773	0,10465116	0,12775657	0,868	5540	tags=26%, list=16%, signal=31%
OCT1_03	220	0,2588611	1,2454007	0,051282052	0,12936427	0,871	5998	tags=25%, list=18%, signal=30%
DR4_Q2	246	0,27091184	1,2435992	0,0729927	0,13036707	0,872	6248	tags=27%, list=18%, signal=33%
AP1_01	252	0,28486508	1,242418	0,11186441	0,13119318	0,873	5540	tags=25%, list=16%, signal=29%
STAT6_02	249	0,27417165	1,2404056	0,07324841	0,13241121	0,873	8036	tags=35%, list=24%, signal=45%
ACCTGTTG_UNKNOWN	148	0,2774346	1,2393173	0,09090909	0,13285413	0,874	6497	tags=28%, list=19%, signal=34%
IK2_01	258	0,26758102	1,2379974	0,076677315	0,1336133	0,875	8228	tags=33%, list=24%, signal=43%
PBX1_01	235	0,26417026	1,2310383	0,09440559	0,13910101	0,879	7269	tags=30%, list=21%, signal=38%
MMEF2_Q6	261	0,2644796	1,2282723	0,12063492	0,14131677	0,882	7120	tags=30%, list=21%, signal=37%
CEBPDELTA_Q6	226	0,26226023	1,2267165	0,07171315	0,14237757	0,883	8605	tags=35%, list=25%, signal=47%
NFKAPPAB_01	241	0,27203166	1,2221606	0,108552635	0,14617993	0,887	8563	tags=36%, list=25%, signal=48%
FOXO1_02	228	0,2775015	1,2221574	0,07666667	0,1457188	0,887	6784	tags=29%, list=20%, signal=36%
HP1SITEFACTOR_Q6	220	0,27377105	1,2219259	0,0608365	0,1454595	0,887	6937	tags=30%, list=20%, signal=37%
ETS1_B	243	0,2762411	1,2137779	0,11641791	0,15338805	0,896	6481	tags=26%, list=19%, signal=32%
HLF_01	241	0,27425644	1,2082361	0,10344828	0,15811127	0,899	7266	tags=30%, list=21%, signal=38%
MAZR_01	206	0,26227292	1,2080715	0,13719513	0,15777616	0,899	8529	tags=35%, list=25%, signal=47%
AFP1_Q6	249	0,26340497	1,2063766	0,108391605	0,15891273	0,899	7413	tags=31%, list=22%, signal=39%
ZIC1_01	247	0,25964013	1,201772	0,13595167	0,16347417	0,904	8025	tags=32%, list=24%, signal=41%
POU1F1_Q6	228	0,24417153	1,2017162	0,09565217	0,16302341	0,904	7067	tags=29%, list=21%, signal=36%
PAX3_B	84	0,28654867	1,200715	0,15068494	0,16340183	0,905	5278	tags=26%, list=15%, signal=31%
CATRRAGC_UNKNOWN	130	0,25598997	1,1984642	0,1	0,16531882	0,907	6435	tags=26%, list=19%, signal=32%
LMO2COM_01	249	0,24997239	1,1982211	0,106451616	0,16506602	0,907	6296	tags=26%, list=18%, signal=31%
CEBP_Q2_01	259	0,27044225	1,1979898	0,10144927	0,1648291	0,907	8710	tags=37%, list=26%, signal=50%
E2F_Q2	167	0,3373334	1,1944995	0,2370892	0,16780016	0,908	7602	tags=41%, list=22%, signal=52%
RAAGNYNNCTTY_UNKNOWN	140	0,2715458	1,1944995	0,133829	0,16729167	0,908	7376	tags=31%, list=22%, signal=39%
TAXCREB_02	25	0,34965095	1,1941253	0,17158177	0,16715313	0,909	5194	tags=32%, list=15%, signal=38%
IRF7_01	238	0,2698362	1,1929803	0,15140845	0,16790073	0,91	8252	tags=32%, list=24%, signal=42%
IRF1_Q6	241	0,2420894	1,1929222	0,084	0,16744691	0,91	6198	tags=24%, list=18%, signal=29%
GAANYNYGACNY_UNKNOWN	73	0,30127704	1,1920195	0,17801048	0,16789435	0,911	6454	tags=30%, list=19%, signal=37%
CP2_02	239	0,2674088	1,1902157	0,14848486	0,16952443	0,914	6240	tags=28%, list=18%, signal=34%
PIT1_Q6	224	0,25385723	1,190067	0,12416107	0,16920651	0,914	6497	tags=23%, list=19%, signal=28%
HFH3_01	178	0,25866267	1,1895366	0,092369474	0,16918324	0,914	7749	tags=30%, list=23%, signal=39%
SREBP1_02	84	0,2826094	1,1892757	0,16666667	0,16889319	0,914	5160	tags=25%, list=15%, signal=29%
IK3_01	216	0,2617127	1,1885414	0,15789473	0,16914643	0,914	7141	tags=29%, list=21%, signal=37%
MAF_Q6	244	0,26204202	1,1863949	0,125	0,17104867	0,914	6225	tags=25%, list=18%, signal=31%
GRE_C	120	0,27125877	1,1844915	0,14328358	0,17287874	0,919	8036	tags=38%, list=24%, signal=49%
HSF2_01	235	0,24771757	1,1834692	0,114754096	0,17333323	0,919	8841	tags=35%, list=26%, signal=47%
RYTGCNWTGGNR_UNKNOWN	111	0,26349312	1,1809583	0,18897638	0,17577742	0,919	8188	tags=32%, list=24%, signal=43%
PAX8_01	37	0,32886773	1,180909	0,21568628	0,17529169	0,919	3057	tags=22%, list=9%, signal=24%
YKACATTT_UNKNOWN	272	0,25768325	1,1802236	0,11824324	0,17560488	0,919	6797	tags=28%, list=20%, signal=34%
TATA_C	263	0,24431573	1,1777607	0,12648222	0,17813699	0,921	6326	tags=24%, list=19%, signal=29%
ICSBP_Q6	236	0,27400336	1,1773713	0,15488215	0,17811939	0,921	8457	tags=34%, list=25%, signal=45%
ATF3_Q6	231	0,2646261	1,1771675	0,1863354	0,1778661	0,921	7517	tags=29%, list=22%, signal=37%
PEA3_Q6	250	0,29226395	1,1742665	0,21082622	0,18079913	0,922	5229	tags=28%, list=15%, signal=33%
OCT_Q6	247	0,24140614	1,1742018	0,12062257	0,18033344	0,922	4167	tags=16%, list=12%, signal=18%
OCT1_02	203	0,24118282	1,1737387	0,118773945	0,18036608	0,922	6858	tags=24%, list=20%, signal=29%
PPARA_01	37	0,3524692	1,1727693	0,21333334	0,18096927	0,924	3523	tags=24%, list=10%, signal=27%
STAT5B_01	235	0,24039325	1,1712455	0,09417041	0,18215655	0,924	7286	tags=26%, list=21%, signal=33%
TTF1_Q6	249	0,2528732	1,1711729	0,14906833	0,1817474	0,924	6744	tags=26%, list=20%, signal=32%
CART1_01	215	0,23879388	1,1709075	0,13703704	0,18146943	0,924	8538	tags=30%, list=25%, signal=40%
HFH8_01	188	0,24340601	1,1676824	0,11111111	0,18457663	0,924	7781	tags=30%, list=23%, signal=39%
MZF1_01	222	0,25202435	1,1652725	0,19354838	0,18701118	0,927	7005	tags=29%, list=21%, signal=37%
CTCNANGTGNY_UNKNOWN	84	0,28891847	1,1628598	0,2320917	0,18909517	0,928	9555	tags=45%, list=28%, signal=63%
FOXM1_01	239	0,24927561	1,1576985	0,15302491	0,19451354	0,93	7547	tags=29%, list=22%, signal=37%
COREBINDINGFACTOR_Q6	255	0,2495216	1,1560664	0,17966102	0,19628894	0,933	7568	tags=32%, list=22%, signal=41%
TTGCWCAAY_CEBPB_02	59	0,32562613	1,1484321	0,2506812	0,20596321	0,942	7408	tags=37%, list=22%, signal=48%
HFH1_01	230	0,23491064	1,1477956	0,11557789	0,20619005	0,942	5383	tags=21%, list=16%, signal=25%
YRCCAKNNGNCGC_UNKNOWN	63	0,3012208	1,1469128	0,25561798	0,20684145	0,943	6866	tags=32%, list=20%, signal=40%
ETF_Q6	108	0,33499607	1,1446038	0,29383886	0,20970552	0,943	4316	tags=27%, list=13%, signal=31%
PAX8_B	102	0,26542476	1,1439841	0,21246459	0,21003844	0,943	3655	tags=17%, list=11%, signal=19%
FOXJ2_02	230	0,24944913	1,1437365	0,16370107	0,20972359	0,943	7765	tags=29%, list=23%, signal=37%
PR_Q2	248	0,23508584	1,1429427	0,13978495	0,20994245	0,943	6103	tags=23%, list=18%, signal=28%
FREAC3_01	238	0,237277	1,1404052	0,15686275	0,21261011	0,943	6684	tags=26%, list=20%, signal=32%
SRY_02	244	0,25108042	1,1400263	0,18637992	0,21255901	0,943	5874	tags=24%, list=17%, signal=29%
PXR_Q2	247	0,2345513	1,1397184	0,14965986	0,2124069	0,943	4711	tags=19%, list=14%, signal=22%
CIZ_01	222	0,24581704	1,1397022	0,15666667	0,21184435	0,943	8985	tags=37%, list=26%, signal=50%
VDR_Q6	261	0,23722176	1,1395487	0,13986014	0,21144946	0,943	6296	tags=26%, list=18%, signal=32%
ATF6_01	116	0,2671484	1,1378999	0,20504732	0,21299608	0,944	7777	tags=40%, list=23%, signal=51%
HNF4_DR1_Q3	248	0,25279137	1,1336172	0,2	0,21794777	0,947	6622	tags=25%, list=19%, signal=31%
VDR_Q3	209	0,24138	1,1311474	0,18373494	0,22082679	0,949	8574	tags=33%, list=25%, signal=44%
STAT5A_03	249	0,24782616	1,1305293	0,17605634	0,22108358	0,949	8271	tags=32%, list=24%, signal=42%
ACAWYAAAG_UNKNOWN	96	0,27281937	1,128122	0,27197802	0,22353786	0,949	6548	tags=26%, list=19%, signal=32%
HNF3ALPHA_Q6	201	0,2335715	1,1263342	0,14537445	0,22513296	0,95	5383	tags=22%, list=16%, signal=26%
LBP1_Q6	213	0,2591915	1,1240983	0,20833333	0,22762892	0,95	6267	tags=28%, list=18%, signal=34%
CYTAGCAAY_UNKNOWN	139	0,276323	1,1226553	0,26683939	0,22901334	0,95	7793	tags=37%, list=23%, signal=47%
TFIII_Q6	200	0,24587594	1,1222606	0,22751322	0,22897635	0,95	6011	tags=22%, list=18%, signal=27%
NKX25_01	118	0,25070462	1,1217344	0,20967741	0,22916287	0,95	5505	tags=25%, list=16%, signal=29%
GTTRYCATRR_UNKNOWN	161	0,26011708	1,1213477	0,2631579	0,22907987	0,95	8248	tags=34%, list=24%, signal=45%
CCTNTMAGA_UNKNOWN	119	0,2484727	1,1202391	0,2324159	0,23005211	0,951	3965	tags=18%, list=12%, signal=21%
NFKB_C	253	0,2421915	1,119032	0,23131673	0,23130292	0,952	7138	tags=29%, list=21%, signal=36%
ELF1_Q6	228	0,24777836	1,1153686	0,24172185	0,23604476	0,954	8478	tags=37%, list=25%, signal=49%
LFA1_Q6	234	0,23790438	1,1145123	0,2651515	0,23656268	0,954	6528	tags=26%, list=19%, signal=32%
CCCNNGGGAR_OLF1_01	304	0,22037266	1,1133938	0,17721519	0,23767391	0,957	6545	tags=25%, list=19%, signal=31%
AML1_01	253	0,2616023	1,112445	0,26567164	0,23845375	0,958	6899	tags=32%, list=20%, signal=40%
AML1_Q6	253	0,2616023	1,112445	0,26567164	0,23784234	0,958	6899	tags=32%, list=20%, signal=40%
SREBP1_Q6	232	0,2359195	1,1082897	0,25696594	0,2427486	0,96	6240	tags=26%, list=18%, signal=32%

FREAC7_01	182	0,24459796	1,1063373	0,2389706	0,2450296	0,961	7731	tags=29%, list=23%, signal=37%
CEBP_Q3	239	0,23908287	1,1032379	0,20979021	0,24956226	0,962	9084	tags=36%, list=27%, signal=48%
PU1_Q6	216	0,23458265	1,1016061	0,21548821	0,2514435	0,962	8261	tags=31%, list=24%, signal=41%
E47_01	244	0,2442743	1,1015656	0,25739646	0,25085878	0,962	6958	tags=26%, list=20%, signal=33%
MEIS1AHOXA9_01	110	0,24955598	1,1008556	0,2880259	0,25126183	0,962	5674	tags=22%, list=17%, signal=26%
CDPCR3HD_01	224	0,2283019	1,0970784	0,2508591	0,2563429	0,964	7142	tags=26%, list=21%, signal=33%
AR_Q2	119	0,25642258	1,0961981	0,27707008	0,2569673	0,964	8059	tags=36%, list=24%, signal=47%
NFKAPPAB65_01	228	0,23746815	1,0956067	0,2747253	0,2572304	0,964	7373	tags=29%, list=22%, signal=37%
AP1_C	261	0,251385	1,0951269	0,25151515	0,25740957	0,964	8096	tags=30%, list=24%, signal=39%
ZIC3_01	239	0,24158542	1,0918864	0,25364432	0,26190835	0,966	8343	tags=33%, list=24%, signal=43%
GGARNTKYCCA_UNKNOWN	74	0,26320574	1,0913119	0,29545453	0,2623641	0,966	5485	tags=27%, list=16%, signal=32%
RACCACAR_AML_Q6	244	0,23573542	1,0901893	0,25490198	0,2634755	0,966	8211	tags=34%, list=24%, signal=45%
AAANWWTGC_UNKNOWN	188	0,24401742	1,0897516	0,25093633	0,26364166	0,966	9181	tags=35%, list=27%, signal=48%
CCAWWNAAGG_SRF_Q4	84	0,26916197	1,0891691	0,288	0,2638493	0,966	3307	tags=15%, list=10%, signal=17%
ZIC2_01	235	0,24268326	1,0880461	0,2922636	0,26503512	0,966	8042	tags=30%, list=24%, signal=39%
XBP1_01	128	0,26398838	1,0877948	0,2729805	0,26500955	0,967	8977	tags=41%, list=26%, signal=55%
GATA1_02	231	0,2167033	1,0843806	0,20454545	0,26992714	0,97	7204	tags=25%, list=21%, signal=31%
TGTYNNNNNRGCARM_UNKNOWN	82	0,26011148	1,0804073	0,28370786	0,27573466	0,97	7236	tags=32%, list=21%, signal=40%
OCT1_Q6	246	0,22017512	1,0798533	0,2599119	0,2759827	0,97	3717	tags=15%, list=11%, signal=16%
ALX4_01	16	0,3704418	1,0750098	0,34924623	0,28387067	0,97	3710	tags=25%, list=11%, signal=28%
OLF1_01	258	0,22231686	1,0734285	0,2536232	0,28571013	0,97	6102	tags=24%, list=18%, signal=30%
PTF1BETA_Q6	230	0,22601941	1,0730275	0,2599119	0,28573576	0,97	8883	tags=35%, list=26%, signal=47%
IRF1_01	234	0,23272538	1,0717075	0,27561837	0,28722718	0,97	8406	tags=29%, list=25%, signal=38%
NKX3A_01	219	0,22042663	1,0676459	0,2931727	0,29304343	0,97	6018	tags=21%, list=18%, signal=25%
AP4_Q6_01	243	0,21761058	1,0659264	0,27372262	0,29555872	0,971	6102	tags=23%, list=18%, signal=28%
AP1_Q2_01	260	0,23660204	1,064611	0,2862069	0,2969942	0,972	8318	tags=33%, list=24%, signal=43%
ISRE_01	236	0,25521797	1,0645753	0,3213213	0,29632995	0,972	6710	tags=26%, list=20%, signal=32%
FOXD3_01	189	0,21889785	1,0642825	0,27142859	0,29620075	0,972	7677	tags=29%, list=23%, signal=37%
OCT1_B	253	0,21664761	1,0623502	0,28444445	0,29893708	0,973	4108	tags=15%, list=12%, signal=17%
PAX5_01	147	0,24416192	1,062256	0,336	0,29835513	0,973	7682	tags=31%, list=23%, signal=40%
FXR_IR1_Q6	98	0,25390485	1,0622379	0,34181818	0,29766643	0,973	5929	tags=24%, list=17%, signal=30%
HEN1_02	186	0,23046155	1,0582458	0,31230283	0,30431727	0,976	6267	tags=27%, list=18%, signal=33%
RYTAAWNNNTGAY_UNKNOWN	59	0,26584092	1,0578035	0,29900333	0,3043381	0,976	7352	tags=32%, list=22%, signal=41%
CREL_01	246	0,22288133	1,055647	0,32214764	0,30747703	0,976	9381	tags=38%, list=28%, signal=52%
NF1_Q6_01	259	0,22521967	1,0532815	0,3253012	0,3107559	0,976	6514	tags=26%, list=19%, signal=32%
OCT1_Q5_01	252	0,21881332	1,0529045	0,3137255	0,31070423	0,976	4434	tags=16%, list=13%, signal=18%
GTGGGTGK_UNKNOWN	281	0,22240914	1,0524052	0,32269505	0,31070614	0,976	6299	tags=26%, list=18%, signal=31%
SRF_Q6	232	0,23029265	1,052185	0,33898306	0,31034014	0,976	4196	tags=16%, list=12%, signal=19%
NF1_Q6	248	0,22583562	1,0510691	0,3320158	0,3116082	0,976	8802	tags=35%, list=26%, signal=46%
TEF1_Q6	212	0,24492407	1,0497926	0,35327634	0,31343907	0,976	6652	tags=30%, list=20%, signal=37%
RYTGCNNRGNAAC_MIF1_01	81	0,2860447	1,0496044	0,35466668	0,3129802	0,976	7823	tags=33%, list=23%, signal=43%
RGAGGAARY_PU1_Q6	473	0,21180443	1,0469564	0,30451128	0,31760904	0,977	5915	tags=23%, list=17%, signal=27%
POU6F1_01	225	0,22813351	1,0462224	0,3576389	0,31835526	0,977	6223	tags=23%, list=18%, signal=28%
LEF1_Q2	210	0,2454928	1,0451621	0,34615386	0,3196957	0,977	6373	tags=25%, list=19%, signal=31%
FREAC2_01	248	0,22578038	1,0448235	0,34146342	0,3195029	0,977	8122	tags=31%, list=24%, signal=41%
AP2REP_01	170	0,2240454	1,0422814	0,34076434	0,32355267	0,978	5388	tags=22%, list=16%, signal=26%
YATGNWAAT_OCT_C	342	0,20226769	1,0419337	0,342723	0,32359385	0,978	4586	tags=16%, list=13%, signal=18%
ETS2_B	264	0,22584178	1,0391728	0,32592592	0,32852173	0,98	8162	tags=33%, list=24%, signal=42%
FOX_Q2	204	0,21634707	1,0390934	0,3425926	0,32786852	0,98	6384	tags=23%, list=19%, signal=28%
MYOGENIN_Q6	241	0,2222302	1,0385431	0,33427763	0,32830617	0,98	4820	tags=19%, list=14%, signal=22%
OCT_C	247	0,2093035	1,0380496	0,33333334	0,3286088	0,98	3942	tags=15%, list=12%, signal=16%
ARGGGTTAA_UNKNOWN	117	0,24892627	1,035087	0,371875	0,33440065	0,983	7352	tags=29%, list=22%, signal=37%
STTTCRNTTT_IRF_Q6	175	0,2590402	1,0345436	0,3799392	0,3347089	0,983	6687	tags=23%, list=20%, signal=29%
YGACNNYACAR_UNKNOWN	93	0,2301392	1,0326535	0,3933518	0,33761716	0,984	5814	tags=23%, list=17%, signal=27%
OCT1_01	252	0,20892532	1,030161	0,3690476	0,34204978	0,985	3942	tags=14%, list=12%, signal=16%
OCT1_05	242	0,20834634	1,0292224	0,37547892	0,3432416	0,985	4605	tags=17%, list=14%, signal=19%
RTTTNNNYTGGM_UNKNOWN	144	0,2348197	1,0275623	0,40273038	0,34584758	0,985	7682	tags=30%, list=23%, signal=38%
NKX25_02	253	0,21567632	1,0274059	0,3846154	0,34531832	0,985	6733	tags=25%, list=20%, signal=31%
HOXA4_Q2	256	0,2145605	1,0264221	0,3409091	0,34646225	0,986	8574	tags=33%, list=25%, signal=44%
IRF_Q6	229	0,23809855	1,0203162	0,41230768	0,35775733	0,986	6935	tags=27%, list=20%, signal=34%
HNF6_Q6	227	0,20669623	1,0199122	0,40585774	0,35781628	0,986	8550	tags=26%, list=25%, signal=35%
HTF_01	67	0,27326074	1,0172802	0,42670158	0,36241132	0,987	8973	tags=48%, list=26%, signal=65%
CDPCR1_01	125	0,21546793	1,0128286	0,40604028	0,37131232	0,988	5388	tags=22%, list=16%, signal=26%
MEF2_04	24	0,2853955	1,007645	0,46285716	0,3808211	0,989	6090	tags=29%, list=18%, signal=35%
HNF4ALPHA_Q6	259	0,23301826	1,0020218	0,44107744	0,39291072	0,989	6453	tags=25%, list=19%, signal=30%
TAAYNRNNTCC_UNKNOWN	165	0,20929372	1,0005611	0,48253968	0,39506435	0,989	6435	tags=22%, list=19%, signal=27%
CCANNAGRKGGC_UNKNOWN	111	0,23479185	0,9994737	0,45938376	0,39659807	0,989	8800	tags=37%, list=26%, signal=50%
EVI1_02	126	0,21084675	0,9977419	0,4720812	0,39998618	0,989	6387	tags=24%, list=19%, signal=29%
GFI1_01	255	0,20062989	0,99393165	0,45909092	0,4073872	0,99	6443	tags=23%, list=19%, signal=28%
TAL1BETAITF2_01	246	0,19989982	0,9937816	0,48557693	0,4069846	0,99	5970	tags=21%, list=18%, signal=25%
IPF1_Q4	240	0,20699848	0,9887185	0,4979757	0,4176313	0,993	7839	tags=27%, list=23%, signal=34%
AML_Q6	252	0,21431114	0,98853695	0,45	0,41705006	0,993	8074	tags=32%, list=24%, signal=41%
AP3_Q6	232	0,20609939	0,9882245	0,48249027	0,41715422	0,994	6231	tags=22%, list=18%, signal=27%
S8_01	239	0,19883256	0,9870722	0,4367347	0,41903573	0,994	4402	tags=16%, list=13%, signal=18%
GGGNNTTTCC_NFKB_Q6_01	131	0,23601516	0,98667973	0,47398844	0,41882312	0,994	7684	tags=29%, list=23%, signal=37%
EN1_01	106	0,22358255	0,9860656	0,50769234	0,41927746	0,994	7906	tags=32%, list=23%, signal=42%
CEBP_01	259	0,19812569	0,9781696	0,5020243	0,43614003	0,994	8347	tags=30%, list=24%, signal=40%
LEF1_Q6	242	0,20823474	0,9710414	0,49027237	0,45160112	0,994	5932	tags=23%, list=17%, signal=28%
NKX61_01	226	0,19715236	0,96914774	0,53881276	0,45544323	0,994	6932	tags=23%, list=20%, signal=29%
CTGRYYYNATT_UNKNOWN	66	0,23095933	0,96839607	0,5154062	0,45631373	0,994	4843	tags=17%, list=14%, signal=19%
MEIS1BHOXA9_01	135	0,20164424	0,9661319	0,57706094	0,4605936	0,994	8340	tags=30%, list=24%, signal=39%
EVI1_04	228	0,20474443	0,96322745	0,5762082	0,46658906	0,994	7731	tags=29%, list=23%, signal=37%
MEF2_01	137	0,21116915	0,96143746	0,5808581	0,46991292	0,994	6549	tags=26%, list=19%, signal=31%
AP4_Q5	259	0,196908	0,9607055	0,54208755	0,4705421	0,994	6544	tags=23%, list=19%, signal=28%
STAT4_01	254	0,2007273	0,95696187	0,5931559	0,47871813	0,994	8036	tags=29%, list=24%, signal=38%
P53_02	242	0,20740798	0,9552825	0,58064514	0,48221087	0,994	8046	tags=29%, list=24%, signal=37%
SRF_Q5_01	212	0,2119892	0,95413864	0,50308645	0,48388582	0,994	6650	tags=22%, list=19%, signal=27%
CTAWWWATA_RSRFC4_Q2	346	0,191928	0,9510599	0,56692916	0,4908051	0,995	8503	tags=29%, list=25%, signal=38%
CDP_02	104	0,2106235	0,9501984	0,54651165	0,49197343	0,995	5994	tags=19%, list=18%, signal=23%
CDP_01	84	0,22377476	0,94749826	0,59786475	0,49835774	0,996	5556	tags=20%, list=16%, signal=24%
TAL1ALPHAE47_01	238	0,19085017	0,942103	0,6111111	0,51117617	0,996	6038	tags=20%, list=18%, signal=24%
MYOD_Q6	237	0,17847991	0,93060374	0,6871795	0,54020566	0,997	5141	tags=17%, list=15%, signal=20%
TGGNNNNNNKCCAR_UNKNOWN	393	0,18795118	0,92501974	0,6869159	0,55380225	0,998	6527	tags=24%, list=19%, signal=29%
HNF3_Q6	184	0,19534974	0,921927	0,73170733	0,56034636	0,998	7685	tags=27%, list=23%, signal=34%
HAND1E47_01	260	0,18831822	0,92168653	0,65975106	0,55970216	0,998	6908	tags=23%, list=20%, signal=29%
HNF4_01	251	0,22264814	0,9199235	0,56285715	0,562918	0,998	5609	tags=22%, list=16%, signal=26%
HNF4_Q6	242	0,20519911	0,91357756	0,6636364	0,5786335	0,998	4169	tags=17%, list=12%, signal=19%
AAAYWAACM_HFH4_01	241	0,19503583	0,91320187	0,74336284	0,57847023	0,998	6933	tags=24%, list=20%, signal=30%
DBP_Q6	243	0,19012724	0,9083108	0,7613636	0,58990234	0,998	5160	tags=19%, list=15%, signal=22%
WGGAATGY_TEF1_Q6	353	0,19029573	0,9058559	0,6989619	0,59496695	0,998	6175	tags=23%, list=18%, signal=27%
CDC5_01	235	0,19162183	0,90564334	0,7194245	0,5944839	0,998	7236	tags=25%, list=21%, signal=31%
HNF4_01_B	239	0,21502317	0,9052546	0,6147541	0,59434533	0,998	8266	tags=31%, list=24%, signal=40%
TAAWWATAG_RSRFC4_Q2	159	0,19733873	0,89458084	0,71428573	0,62125546	0,998	7786	tags=28%, list=23%, signal=36%
MEF2_Q6_01	235	0,18896261	0,8936863	0,734375	0,62237495	0,998	7444	tags=26%, list=22%, signal=34%
SRF_Q4	215	0,19515646	0,8919909	0,6984127	0,62550616	0,998	5814	tags=20%, list=17%, signal=25%
E47_02	235	0,18139052	0,8899367	0,80444443	0,62928945	0,998	5079	tags=17%, list=15%, signal=20%
SRY_01	215	0,20687103	0,88934034	0,6594203	0,62971175	0,998	5020	tags=20%, list=15%, signal=24%
HEN1_01	187	0,19059129	0,88522106	0,73397434	0,6388654	0,998	7371	tags=28%, list=22%, signal=36%
E2A_Q2	232	0,18542624	0,88283825	0,77272725	0,6434849	0,998	4779	tags=17%, list=14%, signal=19%
SRF_C	207	0,19349088	0,87503844	0,71568626	0,66200966	0,998	7195	tags=23%, list=21%, signal=29%
RSRFC4_Q2	206	0,17853703	0,8742006	0,7975708	0,6627794	0,998	7731	tags=27%, list=23%, signal=34%
GATA1_03	235	0,16976899	0,87165564	0,8277512	0,66812444	0,998	7984	tags=27%, list=23%, signal=35%
HNF3B_01	204	0,17463338	0,8591253	0,8469945	0,69627756	0,998	6858	tags=22%, list=20%, signal=27%
AREB6_01	259	0,17714591	0,8539458	0,8101266	0,7072073	0,998	6714	tags=23%, list=20%, signal=29%
WYAAANNRNNNGCG_UNKNOWN	61	0,21181558	0,8519463	0,71099746	0,7105491	0,998	7492	tags=31%, list=22%, signal=40%
PAX2_02	246	0,17879103	0,8495672	0,8582996	0,71481115	0,998	7352	tags=25%, list=22%, signal=32%
YTAAYNGCT_UNKNOWN	145	0,17391287	0,8472718	0,88601035	0,71840096	0,998	6937	tags=23%, list=20%, signal=29%
TGATTTRY_GFI1_01	283	0,1724305	0,84550166	0,8691983	0,7208901	0,998	5904	tags=18%, list=17%, signal=22%
P53_DECAMER_Q2	244	0,17849867	0,8438109	0,8924731	0,7231647	0,998	8042	tags=27%, list=24%, signal=36%
GATA3_01	230	0,16750346	0,84356236	0,869403	0,7223017	0,998	7517	tags=25%, list=22%, signal=32%
YTAATTAA_LHX3_01	177	0,1662216	0,829552	0,9018868	0,7548632	0,998	3445	tags=11%, list=10%, signal=13%
LMO2COM_02	232	0,16886543	0,82945883	0,91769546	0,7535504	0,998	7228	tags=23%, list=21%, signal=29%
HOX13_01	45	0,22070648	0,82400775	0,75399363	0,7637736	0,998	8576	tags=38%, list=25%, signal=50%
MEF2_03	228	0,17023923	0,8221313	0,91791046	0,7662063	0,998	7786	tags=25%, list=23%, signal=33%
RNCTANNRNNYNATTW_UNKNOWN	62	0,19599639	0,8121723	0,7742947	0,7852498	0,998	6435	tags=24%, list=19%, signal=30%
NKX62_Q2	232	0,15840016	0,7986753	0,94052047	0,8108874	0,999	6684	tags=20%, list=20%, signal=24%
TTANWNANTGGM_UNKNOWN	54	0,19421937	0,7873147	0,87	0,82894766	0,999	7305	tags=24%, list=21%, signal=31%
HMEF2_Q6	132	0,17369808	0,78443974	0,9283582	0,83148044	0,999	6549	tags=22%, list=19%, signal=27%
GNCF_01	71	0,18253715	0,7374008	0,9453125	0,8964749	1	3890	tags=13%, list=11%, signal=14%
SRF_01	50	0,19537605	0,72651803	0,8959391	0,9076009	1	8774	tags=30%, list=26%, signal=40%

Supplementary Table 4

E2F targets genes

AK2	DUT	MYBL2	RBBP7
ANP32E	E2F8	MYC	RFC1
ASF1A	EED	NAA38	RFC2
ASF1B	EIF2S1	NAP1L1	RFC3
ATAD2	ESPL1	NASP	RNASEH2A
AURKA	EXOSC8	NBN	RPA1
AURKB	EZH2	NCAPD2	RPA2
BARD1	GINS1	NOLC1	RPA3
BIRC5	GINS3	NOP56	RQCD1
BRCA1	GINS4	NUDT21	RRM2
BRCA2	GSPT1	NUP107	SHMT1
BRMS1L	H2AFX	NUP153	SLBP
BUB1B	H2AFZ	NUP205	SMC1A
CBX5	HELLS	ORC2	SMC3
CCNB2	HMGA1	ORC6	SMC4
CCNE1	HMGB2	PA2G4	SMC6
CCP110	HMGB3	PAICS	SNRPB
CDC20	HMMR	PAN2	SPAG5
CDC25A	HN1	PCNA	SPC24
CDC25B	HNRNPD	PDS5B	SPC25
CDCA3	HUS1	PHF5A	SRSF1
CDCA8	ILF3	PLK1	SRSF2
CDK1	ING3	PLK4	SSRP1
CDK4	IPO7	PMS2	STAG1
CDKN1A	KIF18B	PNN	STMN1
CDKN1B	KIF22	POLA2	SUV39H1
CDKN2A	KIF2C	POLD1	SYNCRIP
CDKN2C	KIF4A	POLD2	TACC3
CDKN3	KPNA2	POLD3	TBRG4
CENPE	LBR	POLE	TFRC
CENPM	LIG1	POLE4	TIMELESS
CHEK1	LMNB1	POP7	TIPIN
CHEK2	LUC7L3	PPM1D	TK1
CIT	LYAR	PPP1R8	TMPO
CKS1B	MAD2L1	PRDX4	TOP2A

CKS2	MCM2	PRIM2	TP53
CSE1L	MCM3	PRKDC	TRA2B
CTCF	MCM4	PRPS1	TRIP13
DCK	MCM5	PSIP1	TUBG1
DCLRE1B	MCM6	PSMC3IP	UBE2S
DCTPP1	MCM7	PTTG1	UBE2T
DDX39A	MELK	RACGAP1	UBR7
DEK	MKI67	RAD1	UNG
DEPDC1	MLH1	RAD21	USP1
DIAPH3	MMS22L	RAD50	WDR90
DLGAP5	MRE11A	RAD51AP1	WEE1
DNMT1	MSH2	RAD51C	XPO1
DONSON	MTHFD2	RAN	XRCC6
DSCC1	MXD3	RANBP1	ZW10

Supplementary Table 5

Targets of E2F downregulated after treatment with ly101-4B

Gene name	Fold change	p-value
AK2	-0.5216	0.0034
ANP32E	-0.7789	0.0065
ASF1B	-1.4138	0.0455
ATAD2	-1.7596	0.0567
AURKA	-0.5405	0.0034
AURKB	-1.3857	0.0141
BARD1	-1.0164	0.0267
BIRC5	-0.9123	0.0156
BRCA1	-1.0826	0.1079
BRMS1	-0.3470	0.0027
BUB1B	-1.5100	0.0272
CBX5	-0.9429	0.0086
CCNB2	-0.7425	0.0009
CCNE1	-0.7112	0.0367
CDC20	-0.6938	0.0063
CDC25A	-1.3849	0.0116
CDC25B	-0.5561	0.0028
CDCA3	-1.4272	0.0029
CDCA8	-1.1784	0.0322
CDK4	-0.3041	0.0002
CDKN3	-0.6380	0.0111
CENPE	-0.9917	0.0984
CENPM	-0.6976	0.0063
CHEK1	-0.5359	0.0021
CHEK2	-0.7301	0.0001
CIT	-0.9113	0.0013
CKS1B	-0.4637	0.0028
CSE1L	-0.7440	0.0124
DCK	-0.8216	0.0003
DCLRE1B	-1.0169	0.0337
DDX39	-0.5077	0.0031
DNMT1	-1.3456	0.0031
DONSON	-0.4004	0.0039
DSCC1	-1.7005	0.0625
DUT	-0.8181	0.0135
E2F8	-1.9312	0.0693
EED	-0.9729	0.0012
EIF2S1	-0.3895	0.0156
ESPL1	-0.9645	0.0107
EZH2	-0.2165	0.0015
GINS1	-1.9070	0.0184

GINS3	-0.7498	0.0233
GINS4	-1.5148	0.0232
GSPT1	-0.2333	0.0040
H2AFX	-0.7109	0.0023
H2AFZ	-0.6049	0.0051
HELLS	-2.0670	0.0391
HMGB2	-0.8330	0.0026
HMMR	-0.8213	0.0461
HN1	-0.6810	0.0082
HNRNPD	-0.7705	0.0017
HUS1	-0.1938	0.0004
ILF3	-0.5562	0.0013
KIF18B	-0.8887	0.0098
KIF22	-1.2195	0.0009
KIF2C	-0.7321	0.0133
KIF4A	-0.9167	0.0081
KPNA2	-0.8818	0.0019
LBR	-0.4741	0.0100
LIG1	-1.1900	0.0001
LMNB1	-1.6313	0.0009
LYAR	-1.2862	0.0324
MAD2L1	-1.4841	0.0050
MCM2	-2.0835	0.0045
MCM3	-2.0329	0.0112
MCM4	-2.1492	0.0213
MCM5	-2.3212	0.0942
MCM6	-1.1738	0.0222
MCM7	-1.8116	0.0031
MELK	-0.9150	0.0097
MKI67	-1.4136	0.0059
MRE11A	-1.0163	0.0053
MSH2	-1.2998	0.0200
MYBL2	-1.2218	0.0025
NASP	-1.2597	0.0163
NCAPD2	-0.8201	0.0002
NOLC1	-0.8703	0.0087
NUDT21	-0.3899	0.0087
NUP107	-1.0796	0.0038
NUP153	-0.6394	0.0008
NUP205	-1.0003	0.0023
PA2G4	-0.5580	0.0004
PAICS	-0.7243	0.0001
PCNA	-1.7914	0.0018
PDS5B	-0.7371	0.0348
PHF5A	-0.3921	0.0022
PLK1	-0.9347	0.0057
PLK4	-1.9089	0.1552
PNN	-1.0889	0.0499
POLA2	-1.7341	0.0127

POLD1	-1.0791	0.0079
POLD2	-0.3671	0.0002
POLD3	-1.4186	0.0176
POLE	-1.2363	0.0133
POLE4	-0.3786	0.0105
POP7	-0.4187	0.0017
PPM1D	-0.1568	0.0000
PPP1R8	-0.5220	0.0011
PRDX4	-0.0325	0.0000
PRIM2	-1.1799	0.0302
PRKDC	-0.3833	0.0011
PRPS1	-0.8831	0.0022
PSIP1	-0.6192	0.0137
PSMC3IP	-1.2966	0.0622
PTTG1	-0.7056	0.0010
RACGAP1	-0.8624	0.0086
RAD1	-0.4008	0.0043
RAD21	-0.6528	0.0074
RAD23B	-0.1441	0.0012
RAD51AP1	-2.0406	0.1181
RAD51C	-0.7505	0.0494
RAN	-0.2965	0.0055
RANBP1	-1.0762	0.0019
RBBP7	-0.5890	0.0005
RFC2	-1.4370	0.0027
RFC3	-1.9485	0.0473
RNASEH2A	-1.2002	0.0047
RPA1	-0.9290	0.0016
RPA2	-0.7207	0.0167
RQCD1	-0.1227	0.0016
RRM2	-1.4572	0.0160
SHMT1	-0.8976	0.0362
SLBP	-0.5650	0.0005
SMC1A	-1.1972	0.0238
SMC3	-0.9438	0.0773
SMC4	-1.2316	0.0572
SNRPB	-0.4255	0.0092
SPAG5	-1.3103	0.0044
SPC24	-1.8585	0.0002
SPC25	-1.8934	0.0409
SSRP1	-0.4519	0.0045
STMN1	-0.8010	0.0031
SUV39H1	-1.5774	0.0011
SYNCRIP	-0.6138	0.0120
TACC3	-1.2113	0.0037
TBRG4	-0.3837	0.0093
TFRC	-0.2053	0.0010
TIMELESS	-1.1600	0.0023
TIPIN	-1.2558	0.0026

TK1	-1.4830	0.0113
TMPO	-1.2283	0.0191
TOP2A	-1.3845	0.0101
TRA2A	-0.2842	0.0056
TRIP13	-1.1061	0.0101
TUBG1	-0.5008	0.0059
UBE2S	-0.2270	0.0053
UBE2T	-1.2784	0.0798
UNG	-1.6282	0.0022
WEE1	-0.7167	0.0052
XPO1	-0.5274	0.0126
XRCC6	-0.3990	0.0015
